# The effects of prenatal multiple micronutrient supplementation and small-quantity lipid-based nutrient supplementation on small vulnerable newborn types in low-income and middle-income countries: a meta-analysis of individual participant data

**DOI:** 10.1016/S2214-109X(24)00449-2

**Published:** 2025-01-29

**Authors:** Dongqing Wang, Enju Liu, Nandita Perumal, Uttara Partap, Ilana R Cliffer, Janaína Calu Costa, Molin Wang, Wafaie W Fawzi, Gestational Weight Gain Pooling Project Consortium, Seth Adu-Afarwuah, Per Ashorn, Ulla Ashorn, Malay Kanti Mridha, Shams Arifeen, Zulfiqar A Bhutta, Yue Cheng, Parul Christian, Anthony M Costello, Kathryn G Dewey, Henrik Friis, Exnevia Gomo, Rebecca Grais, Ousmane Guindo, Nancy F Krebs, Lieven Huybregts, Sheila Isanaka, Carl Lachat, Anna Lartey, Steven C LeClerq, Kenneth Maleta, Dharma S Manandhar, Reynaldo Martorell, Susana L Matias, Elizabeth M McClure, Sophie E Moore, David Osrin, Willy Urassa, Andrea B Pembe, Andrew M Prentice, Usha Ramakrishnan, Juan Rivera, Arjumand Rizvi, Dominique Roberfroid, Abu Ahmed Shamim, Sajid Soofi, Kerry Schulze, Keith P West Jr, Lee Wu, Lingxia Zeng, Zhonghai Zhu

**Affiliations:** a Department of Global and Community Health, College of Public Health, George Mason University, Fairfax, VA, USA; b Institutional Centers for Clinical and Translational Research and Division of Gastroenterology, Hepatology and Nutrition, Boston Children's Hospital, Boston, MA, USA; c Department of Epidemiology and Biostatistics, Arnold School of Public Health, University of South Carolina, Columbia, SC, USA; d Department of Global Health and Population, Harvard TH Chan School of Public Health, Harvard University, Boston, MA, USA; e Department of Epidemiology, Harvard TH Chan School of Public Health, Harvard University, Boston, MA, USA; f Department of Biostatistics, Harvard TH Chan School of Public Health, Harvard University, Boston, MA, USA; g Department of Nutrition, Harvard TH Chan School of Public Health, Harvard University, Boston, MA, USA

## Abstract

**Background:**

Small vulnerable newborn types, defined by combinations of being born too soon or too small, have distinct determinants and health consequences. We aimed to assess the effects of prenatal multiple micronutrient supplementation (MMS) and small-quantity lipid-based nutrient supplementation (SQ-LNS) on small vulnerable newborn types, which are currently unknown.

**Methods:**

In this meta-analysis, individual participant data from randomised controlled trials of MMS and randomised controlled trials of SQ-LNS in low-income and middle-income countries were used. We systematically searched the literature using PubMed, Embase, and Web of Science to identify randomised controlled trials of prenatal nutritional supplementation using MMS or SQ-LNS among pregnant people published between Jan 1, 2000, and Dec 31, 2021. Studies were excluded if they were conducted exclusively among participants selected by pre-existing health conditions, such as anaemia status, HIV infection, or diabetes. We contacted the corresponding authors of all identified studies to seek data contribution. As individual participant data became available, we mapped relevant variables and harmonised the data across studies. Iron and folic acid supplementation was the control group in most studies. Newborns were classified into ten groups through the combinations of preterm or term birth, small, appropriate, and large for gestational age, and low birthweight (LBW) or non-LBW. Newborns were also analysed using a four-group categorisation of preterm or term and LBW or non-LBW. Log-binomial models were used to estimate study-specific risk ratios (RRs), which were pooled using meta-analyses.

**Findings:**

14 randomised controlled trials of MMS (n=42 618; the mean maternal age at study enrolment was 24·3 years [SD 5.6]; 22 086 [51·8%] male neonates and 20 532 [48·2%] female neonates) and four randomised controlled trials of SQ-LNS (n=6246; the mean maternal age at study enrolment was 23·3 years [SD 5·3]; 3137 [50·2%] male neonates and 3109 [49·8%] female neonates) were used. In the ten-group categorisation of small vulnerable newborns, prenatal MMS reduced the risk of preterm–small for gestational age (SGA)–LBW (RR 0·73, 95% CI 0·64–0·84; p=0·0003); preterm–appropriate for gestational age (AGA)–LBW (0·82, 0·74–0·91; p=0·0010); preterm–AGA–non-LBW (0·89, 0·80–0·98; p=0·019); term–SGA–LBW (0·91, 0·85–0·96; p=0·0046); and term–SGA–non-LBW (0·95, 0·90–1·00; p=0·050). In the four-group categorisation, prenatal MMS reduced the risk of preterm–SGA (0·71, 0·62–0·82; p=0·0002) and term–SGA (0·93, 0·89–0·98; p=0·0066). Prenatal SQ-LNS had no significant effects on the risk of giving birth to small vulnerable newborns except for preterm–large for gestational age–non-LBW in the ten-group categorisation (0·78, 0·65–0·94; p=0·023).

**Interpretation:**

Prenatal MMS and SQ-LNS reduce the risk of giving birth to small vulnerable newborns to varying extents, with the greatest magnitude of effects observed for small vulnerable newborn types that confer the greatest neonatal mortality risk. This study underscores the importance of nutritional supplements in prenatal care.

**Funding:**

Bill & Melinda Gates Foundation.

## Introduction

Low birthweight (LBW) is the leading cause of neonatal mortality and a strong predictor of adverse child health and nutrition outcomes throughout life.^1^, ^2^ Despite progress in reducing LBW, its prevalence remains high, particularly in low-income and middle-income countries (LMICs).^1^ An estimated 19·8 million livebirths were of LBW in 2020.^3^ More than 90% of LBW occurs in LMICs, with south Asia accounting for 48% and sub-Saharan Africa 24% of the global burden.^4^ LBW could be attributed to two underlying pathways: short pregnancy duration and fetal growth restriction. Short pregnancy duration is commonly defined by preterm birth, and fetal growth restriction is commonly measured using small for gestational age (SGA) births.

**Research in context**

**Evidence before this study**

We searched PubMed from database inception until April 1, 2022, to identify randomised controlled trials of prenatal multiple micronutrient supplementation (MMS) and small-quantity lipid-based nutrient supplementation (SQ-LNS) on small vulnerable newborns conducted in low-income and middle-income countries. We did not identify any studies that focused on the effect of these nutritional supplements on small vulnerable newborn types. Preterm births, small for gestational age (SGA) births, and low birthweight (LBW) births have been examined as separate constructs. There is little evidence on how prenatal MMS and SQ-LNS affect the risk of giving birth to small vulnerable newborns, an term representing the combinations of adverse birth outcomes that might have distinct determinants, health consequences, and prevention strategies.

**Added value of this study**

This study contributes to a nascent evidence base on the management and intervention strategies of small vulnerable newborn types that combine different adverse birth outcomes that have traditionally been evaluated separately. This study found that prenatal MMS reduced the risk of giving birth to small vulnerable newborns, particularly the small vulnerable newborn types conferring the greatest risk of neonatal mortality, including preterm–SGA–LBW; preterm–appropriate for gestational age–LBW; and preterm–SGA.

**Implications of all the available evidence**

The available evidence cumulatively underscores the importance of nutritional supplements in prenatal care in low-income and middle-income countries. In particular, prenatal MMS had protective effects on most small vulnerable newborn types, which were stronger on the types with the greatest mortality risk, lending robust support for the switch to MMS as the standard antenatal care.

Preterm births, SGA births, and LBW births have traditionally been evaluated as separate outcomes. Attention has recently been paid to the combinations of these adverse birth outcomes that might have distinct distributions, mechanisms, health effects, and management and intervention strategies.^2^, ^5^, ^6^, ^7^, ^8^ Under this framework, preterm birth, SGA births, and LBW births come together under the unified concept of small vulnerable newborn.^2^, ^9^ Of the 135 million livebirths that occurred globally in 2020, 35·3 million (26%) were small vulnerable newborns who were preterm or SGA, including 21·9 million (16%) who were term–SGA, 11·9 million (9%) who were preterm and not SGA, and 1·5 million (1%) who were preterm–SGA.^3^ These vulnerable newborn types have disproportionate burdens of neonatal mortality. Of the 2·4 million neonatal deaths in 2020 globally, 32·8% were attributed to preterm newborns who were not SGA, 14·7% to term–SGA newborns, and 7·7% to preterm–SGA newborns.^3^ Taken together, almost 1·4 million of the 2·4 million neonatal deaths in 2020 were attributable to preterm birth or SGA births.^3^

Antenatal nutritional interventions might reduce the risk of giving birth to small vulnerable newborns. Compared with iron and folic acid supplements, prenatal multiple micronutrient supplementation (MMS), which includes iron and folic acid, reduces the risks of SGA birth and LBW birth and might also prevent preterm birth.^10^, ^11^ WHO currently recommends the use of MMS in the context of rigorous research,^12^ and there have been increasing calls to shift the standard of antenatal care from iron and folic acid to MMS. Prenatal lipid-based nutrient supplements (LNSs) are nutritional supplements that provide not only micronutrients but also energy and essential fatty acids. Small-quantity lipid-based nutrient supplementation (SQ-LNS) uses LNSs that provide around 120 kcal per day and are designed for the prevention of undernutrition.^13^, ^14^ Systematic reviews and meta-analyses showed that prenatal LNSs increase birthweight and reduce the risk of a newborn being SGA.^15^, ^16^

The effects of MMS and SQ-LNS on small vulnerable newborns have not been previously estimated. Identifying the effects of nutritional supplements on small vulnerable newborn types will enhance our understanding of their benefits during pregnancy by ascertaining which small vulnerable newborn types are most affected by such interventions. In this study, we pooled individual-level data from randomised controlled trials to examine the effects of prenatal MMS and SQ-LNS on the risk of giving birth to small vulnerable newborns.

## Methods

### Search strategy and selection criteria

This study was a meta-analysis of individual participant data from randomised controlled trials of prenatal MMS or SQ-LNS in LMICs. We described the design and procedures of this pooling project previously.^17^ Briefly, we systematically searched the literature using PubMed, Embase, and Web of Science to identify randomised controlled trials of prenatal nutritional supplementation using MMS or SQ-LNS among pregnant people published between Jan 1, 2000, and Dec 31, 2021. The PubMed search strategy is provided in the appendix (p 8). There were three inclusion criteria. First, studies had to be randomised controlled trials of prenatal nutritional supplementation using MMS or SQ-LNS. We defined SQ-LNS as LNSs providing less than 120 kcal of energy per day. Second, studies had to be at least partially conducted in a low-income, lower-middle-income, or upper-middle-income economy defined by the World Bank country classification for the 2021 fiscal year. Third, the nutritional supplement could be provided alone or in combination with a co-intervention that was similar across study groups.

We excluded studies conducted exclusively among participants selected by pre-existing health conditions, such as anaemia status, HIV infection, or diabetes. DW and EL reviewed the references of the identified studies and previous systematic reviews to locate additional relevant studies. Two team members independently conducted title and abstract screening and full-text screening, with any discrepancies resolved by discussion with other team members.

We contacted the corresponding authors of all identified studies to seek data contribution. As individual participant data became available, we mapped relevant variables and harmonised the data across studies. An updated search was conducted on May 4, 2024, and no additional eligible studies were identified (appendix p 18). The characteristics of eligible studies that were not included in this analysis due to an absence of individual participant data are summarised in the appendix (p 9).

### Data analysis

Small vulnerable newborns included all live newborns who were preterm (born before 37 completed weeks of gestation), SGA (weight at birth less than the tenth percentile for sex and gestational age based on the INTERGROWTH-21st newborn size standards), or had LBW (weight at birth less than 2500 g).^2^ We classified newborns using a ten-group and a four-group categorisation.^2^, ^6^, ^7^ The ten-group categorisation grouped newborns through the combinations of birth outcomes in three dimensions: (1) term birth (ie, born at or after 37 completed weeks of gestation) and preterm birth; (2) SGA, appropriate for gestational age (AGA; ie, between 10th and 90th percentile for sex and gestational age), and large for gestational age (LGA; ie, greater than 90th percentile for sex and gestational age); and (3) LBW and non-LBW (ie, ≥2500 g). The permutations across the three dimensions yielded ten exhaustive and mutually exclusive combinations. First, term–AGA–non-LBW. Second, term–SGA–non-LBW. Third, term–LGA–non-LBW. Fourth, term–SGA–LBW. Fifth, term–AGA–LBW. Sixth, preterm–SGA–LBW. Seventh, preterm–AGA–LBW. Eighth, preterm–LGA–LBW. Ninth, preterm–AGA–non-LBW. Finally, preterm–LGA–non-LBW. Term–AGA–non-LBW was considered the reference type, whereas the other nine types were considered the small vulnerable groups. Term–LGA–non-LBW does not entail smallness in the dimension of size or gestational age; however, as LGA is associated with increased risks of birth complications and newborn morbidity,^18^ we considered this group in the analysis as a vulnerable newborn type. The four-group categorisation classified newborns into four types. First, term–non-SGA (ie, AGA or LGA). Second, preterm–non-SGA. Third, term–SGA. Finally, preterm–SGA. The term–non-SGA group was considered the reference type.

We used a two-stage analytical approach to obtain estimates from each study and combine the study-specific estimates using meta-analyses.^19^ Within each study, we used log-binomial or modified Poisson models with robust variance estimation^20^ to estimate the effects of prenatal MMS and SQ-LNS on small vulnerable newborn types. We estimated risk ratios (RRs) and 95% CIs comparing MMS or SQ-LNS with each study's control group. We used modified Poisson models with cluster-robust standard errors for cluster-randomised controlled trials.^21^ For studies with multiple interventions (eg, studies with a factorial design), we assessed the statistical interaction between the intervention of interest (ie, prenatal MMS or SQ-LNS) and the additional intervention by including product terms between the two interventions. In the absence of evidence for statistical interaction (p>0·20 for the product term), we combined study groups based on whether prenatal MMS or SQ-LNS were provided. We conducted intention-to-treat analyses. We used fixed-effect and random-effects inverse-variance meta-analyses to pool the study-specific effects. In the random-effects models, we used the Hartung–Knapp–Sidik–Jonkman method, which provides more accurate variance estimates when the number of included studies is low (as in the case for the SQ-LNS studies in this analysis).^22^, ^23^ Missing data were handled using complete case analysis. For the MMS analysis, the percentage of participants with missing outcome data ranged from 9% to 44% across studies, with an overall percentage of 28%; for the SQ-LNS analysis, the percentage ranged from 11% to 22% across studies, with an overall percentage of 18% (appendix p 10). The reasons for missing outcome data were a lack of information on birthweight (22% in the MMS analysis and 12% in the SQ-LNS analysis), followed by a lack of information on gestational age at birth (13% in the MMS analysis and 11% in the SQ-LNS analysis) and a lack of information on infant sex (8% in the MMS analysis and 6% in the SQ-LNS analysis). We used funnel plots to assess the presence of publication bias when three or more studies were included. We did not adjust for multiple comparisons.^24^

We used exploratory subgroup analyses to examine effect modification by (1) maternal age (<20 years, 20–29 years, and ≥30 years); (2) parity (0 and ≥1); (3) gestational age at study enrolment (<20 weeks and ≥20 weeks); (4) maternal early-pregnancy BMI (underweight [ie, BMI <18·5 kg/m^2^], normal weight [ie, BMI 18·5 kg/m^2^ to <25·0 kg/m^2^], and overweight or obesity [ie, BMI ≥25·0 kg/m^2^]);^17^, ^25^ and (5) maternal anaemia at baseline (no anaemia [ie, haemoglobin concentration ≥11·0 g/dL]; mild anaemia [ie, haemoglobin concentration ≥10·0 g/dL and <11·0 g/dL]; and moderate to severe anaemia [ie, haemoglobin concentration <10·0 g/dL]).^26^ In a sensitivity analysis, we adjusted for the continuous form of all these variables as covariates when generating the study-specific estimates. To assess the linearity assumption underlying the log-binomial or modified Poisson models, we inspected the plots of the predicted log probabilities against each continuous predictor. We also inspected the partial residual plots, which showed the partial residuals for each continuous predictor against the values of the corresponding predictor. We did not identify deviation from the linearity assumption. In another sensitivity analysis, we restricted the analysis to studies with ultrasound-based measures of gestational age. We also conducted a secondary analysis on the effects of MMS and SQ-LNS on neonatal mortality, defined as death within the first 28 days of life.

Datasets that were shared for this secondary analysis did not contain personal identifiers and were deemed exempt from requiring ethical approval by the Harvard TH Chan School of Public Health Institutional Review Board. In the original parent studies, maternal consent was obtained, and investigators were covered under individual local ethical approvals. All analyses were conducted using a two-sided α level of 0·05. We conducted the study-specific analyses using SAS 9.4 and the meta-analyses using Comprehensive Meta-Analysis Software version 4.

For randomised controlled trials that were individually randomised, we assessed the risk of bias using the ROB-2 tool.^27^ For cluster-randomised controlled trials, we used the ROB-2 tool for cluster-randomised studies.^28^

### Role of the funding source

The Knowledge Integration initiative at the Bill & Melinda Gates Foundation facilitated the curation and harmonisation of the individual participant data. The funder of the study had no role in study design, data analysis, data interpretation, or writing of the report.

## Results

We identified 19 eligible MMS studies, of which 14 had individual participant data available; we identified four eligible SQ-LNS studies, all of which shared individual participant data (appendix p 18). Therefore, 14 studies were included for the analysis of prenatal MMS, and four studies were included for the analysis of prenatal SQ-LNS. The MMS analysis included 42 618 participants, with 23 292 in the control groups and 19 326 in the MMS groups. The SQ-LNS analysis included 6246 participants, with 3832 in the control groups and 2414 in the SQ-LNS groups. In the MMS analysis, the mean maternal age at study enrolment was 24·34 years (SD 5·60) and included 22 086 (51·8%) male neonates and 20 532 (48·2%) female neonates. In the SQ-LNS analysis, the mean maternal age at study enrolment was 23·32 years (SD 5·33), and included 3137 (50·2%) male neonates and 3109 (49·8%) female neonates. The control group was iron and folic acid supplementation in almost all studies (table 1), except for one MMS study that used iron alone and one multi-country SQ-LNS study that used the standard of antenatal care (which might or might not have included iron and folic acid supplementation) as the control group. Term**–**AGA**–**non-LBW was the most common newborn type based on the ten-group categorisation, accounting for 18 244 (42·8%) of the analytical samples for the MMS analysis and 2493 (39·9%) for the SQ-LNS analysis (table 2). For the four-group categorisation, term**–**non-SGA accounted for most (MMS analysis n=19 344 [45·4%], SQ-LNS analysis n=2621 [42·0%]) of the sample, followed by term**–**SGA (MMS analysis n=16 476 [38·7%], SQ-LNS analysis n=2496 [40·0%]); preterm**–**non-SGA (MMS analysis n=5752 [13·5%], SQ-LNS analysis n=980 [15·7%]); and preterm**–**SGA (MMS analysis n=1046 [2·5%], SQ-LNS analysis n=149 [2·4%]).

**Table 1:** Included randomised controlled trials on MMS and SQ-LNS

	**Country**	**Years of study**	**Sample size in analysis**	**Study design**	**Composition of MMS or SQ-LNS**	**Control group**	**Timing of intervention initiation**	**Gestational age measure**
**MMS studies**								
Christian et al (2003)^29^	Nepal	1998–2001	2383	Cluster	10 μg vitamin D, 10 mg vitamin E, 1·6 mg vitamin B1, 1·8 mg vitamin B2, 20 mg niacin, 2·2 mg vitamin B6, 2·6 μg vitamin B12, 100 mg vitamin C, 65 μg vitamin K, 2·0 mg copper, 100 mg magnesium, 400 μg folic acid, 60 mg iron, 30 mg zinc, and 1000 μg vitamin A, provided daily	Four groups: iron and folic acid* plus 1000 μg vitamin A; iron and folic acid* plus 30 mg zinc plus 1000 μg vitamin A; 400 μg folic acid plus 1000 μg vitamin A; or 1000 μg vitamin A only; all provided daily	From a mean 11 weeks' (SD 5)	LMP
Ramakrishnan et al (2003)^30^	Mexico	1997–2000	636	Parallel	2150 IU vitamin A, 309 IU vitamin D_3_, 5·73 IU vitamin E, 0·93 mg vitamin B1, 1·87 mg vitamin B2, 15·5 mg niacin, 215 μg folic acid, 1·94 mg vitamin B6, 2·04 μg vitamin B12, 66·5 mg vitamin C, 12·9 mg zinc, 62·4 mg iron, and 252 mg magnesium, provided 6 days per week	60 mg per day iron, provided 6 days per week	<13 weeks' gestation	LMP
Friis et al (2004)^31^	Zimbabwe	1996–97	1001	Parallel	3000 μg RE vitamin A, 3·5 mg β-carotene, 1·5 mg vitamin B1, 1·6 mg vitamin B2, 2·2 mg vitamin B6, 4·0 μg vitamin B12, 17 mg niacin, 80 mg vitamin C, 10 μg vitamin D, 10 mg vitamin E, 15 mg zinc, 1·2 μg copper, and 65 μg selenium, plus iron and folic acid provided through standard antenatal care, provided daily	Iron and folic acid provided through standard antenatal care, provided daily	22–36 weeks' gestation	LMP
Osrin et al (2005)^32^	Nepal	2002–04	1004	Parallel	MMS of the UNIMMAP formulation, provided daily	Iron and folic acid,* provided daily	12–20 weeks' gestation	Ultrasound
Fawzi et al (2007)^33^	Tanzania	2001–04	7147	Parallel	20 mg vitamin B1, 20 mg vitamin B2, 25 mg vitamin B6, 100 mg niacin, 50 μg vitamin B12, 500 mg vitamin C, 30 mg vitamin E, 0·8 mg folic acid, plus 60 mg per day iron and 250 μg per day folic acid, provided daily	60 mg per day iron and 250 μg per day folic acid, provided daily	12–27 weeks' gestation	LMP
Zeng et al (2008)^34^	China	2002–06	4264	Cluster	MMS of the UNIMMAP formulation, provided daily	Iron and folic acid* or 400 μg folic acid, provided daily	≤28 weeks' gestation	LMP
Roberfroid et al (2008)^35^	Burkina Faso	2004–06	1020	Factorial	MMS of the UNIMMAP formulation, with chloroquine or sulfadoxine–pyrimethamine, provided daily	Iron and folic acid* with chloroquine or sulfadoxine–pyrimethamine	From a mean 17 weeks (SD 8)	Ultrasound; LMP if ultrasound results are unavailable
Bhutta et al (2009)^36^	Pakistan	2002–04	1319	Cluster	MMS of the UNIMMAP formulation, provided daily	Iron and folic acid,* provided daily	<16 weeks' gestation	Ultrasound and LMP
Persson et al (2012)^37^	Bangladesh	2001–03	3026	Factorial	MMS of the UNIMMAP formulation, provided daily with early or usual initiation of food supplementation	Four groups: 30 mg iron and 400 μg folic acid or 60 mg iron and 400 μg folic acid; provided daily with early or usual initiation of food supplementation	6–8 weeks' gestation	LMP
Moore et al (2012)^38^	The Gambia	2010–12	666	Factorial	60 mg iron, 400 μg folic acid, 1600 μg RE vitamin A, 400 IU vitamin D, 20 mg vitamin E, 140 mg vitamin C, 2·8 mg vitamin B1, 2·8 mg vitamin B2, 36 mg niacin, 2·8 mg vitamin B6, 5·2 μg vitamin B12, 30 mg zinc, 4 mg copper, 130 μg selenium, and 300 μg iodine, provided daily, with or without protein-energy supplements	Iron and folic acid* with or without protein–energy supplements, provided daily	<20 weeks' gestation	Ultrasound
West et al (2014)^39^	Bangladesh	2008–12	17 115	Cluster	770 μg RE vitamin A, 5 μg vitamin D, 15 mg vitamin E, 1·4 mg vitamin B1, 1·4 mg vitamin B2, 18 mg niacin, 1·9 mg vitamin B6, 600 μg folic acid, 2·6 μg vitamin B12, 85 mg vitamin C, 27 mg iron, 12 mg zinc, 1 mg copper, 60 μg selenium, and 220 μg iodine, provided daily	27 mg per day iron and 600 μg per day folic acid, provided daily	Mostly <13 weeks' gestation	LMP
Ashorn et al (2015)^40^	Malawi	2011–13	790	Parallel	800 μg RE vitamin A, 10 μg vitamin D, 20 mg vitamin E, 2·8 mg vitamin B1, 2·8 mg vitamin B2, 36 mg niacin, 7 mg vitamin B5, 3·8 mg vitamin B6, 400 μg folic acid, 5·2 μg vitamin B12, 100 mg vitamin C, 45 μg vitamin K, 20 mg iron, 30 mg zinc, 4 mg copper, 130 μg selenium, 250 μg iodine, and 2·6 mg manganese, provided daily	Iron and folic acid,* provided daily	≤20 weeks' gestation	Ultrasound
Adu-Afarwuah et al (2015)^41^	Ghana	2009–11	727	Parallel	800 μg RE vitamin A, 10 μg vitamin D, 20 mg vitamin E, 2·8 mg vitamin B1, 2·8 mg vitamin B2, 36 mg niacin, 7 mg vitamin B5, 3·8 mg vitamin B6, 400 μg folic acid, 5·2 μg vitamin B12, 100 mg vitamin C, 45 μg vitamin K, 20 mg iron, 30 mg zinc, 4 mg copper, 130 μg selenium, 250 μg iodine, and 2·6 mg manganese, provided daily	Iron and folic acid,* provided daily	≤20 weeks' gestation	Ultrasound
Bliznashka et al, 2022)^42^	Niger	2014–19	1520	Cluster	30 mg iron, 400 μg folic acid, and 20 other micronutrients providing two times the RDA for most micronutrients, provided daily	Iron and folic acid,* provided daily	<30 weeks' gestation	LMP
**SQ-LNS studies**								
Ashorn et al (2015)^40^	Malawi	2011–13	798	Parallel	118 kcal, 2·6 g protein, 10 g fat, 4·59 g linoleic acid, 0·59 g α-linolenic acid, 800 μg RE vitamin A, 10 μg vitamin D, 20 mg vitamin E, 2·8 mg vitamin B1, 2·8 mg vitamin B2, 36 mg niacin, 7 mg vitamin B5, 3·8 mg vitamin B6, 400 μg folic acid, 5·2 μg vitamin B12, 100 mg vitamin C, 45 μg vitamin K, 20 mg iron, 30 mg zinc, 4 mg copper, 280 mg calcium, 190 mg phosphorus, 200 mg potassium, 65 mg magnesium, 130 μg selenium, 250 μg iodine, and 2·6 mg manganese, provided daily	Iron and folic acid,* provided daily	≤20 weeks' gestation	Ultrasound
Adu-Afarwuah et al (2015)^41^	Ghana	2009–11	716	Parallel	118 kcal, 2·6 g protein, 10 g fat, 4·59 g linoleic acid, 0·59 g αlinolenic acid, 800 μg RE vitamin A, 10 μg vitamin D, 20 mg vitamin E, 2·8 mg vitamin B1, 2·8 mg vitamin B2, 36 mg niacin, 7 mg vitamin B5, 3·8 mg vitamin B6, 400 μg folic acid, 5·2 μg vitamin B12, 100 mg vitamin C, 45 μg vitamin K, 20 mg iron, 30 mg zinc, 4 mg copper, 280 mg calcium, 190 mg phosphorus, 200 mg potassium, 65 mg magnesium, 130 μg selenium, 250 μg iodine, and 2·6 mg manganese, provided daily	Iron and folic acid,* provided daily	≤20 weeks' gestation	Ultrasound
Matias et al (2016)^43^	Bangladesh	2011–12	3116	Cluster	118 kcal, 2·6 g protein, 10 g fat, 4·59 g linoleic acid, 0·59 g α-linolenic acid, 800 μg RE vitamin A, 10 μg vitamin D, 20 mg vitamin E, 2·8 mg vitamin B1, 2·8 mg vitamin B2, 36 mg niacin, 7 mg vitamin B5, 3·8 mg vitamin B6, 400 μg folic acid, 5·2 μg vitamin B12, 100 mg vitamin C, 45 μg vitamin K, 20 mg iron, 30 mg zinc, 4 mg copper, 280 mg calcium, 190 mg phosphorus, 200 mg potassium, 65 mg magnesium, 130 μg selenium, 250 μg iodine, and 2·6 mg manganese, provided daily	Iron and folic acid,* provided daily	≤20 weeks' gestation	LMP
Hambidge et al (2019)^44^	Guatemala, India, Pakistan, and DR Congo	2013–17	1616	Parallel	118 kcal, 2·6 g protein, 10 g fat, 4·59 g linoleic acid, 0·59 g α-linolenic acid, 800 μg RE vitamin A, 1000 IU vitamin D_2_, 20 mg vitamin E, 2·8 mg vitamin B1, 2·8 mg vitamin B2, 36 mg niacin, 7 mg vitamin B5, 3·8 mg vitamin B6, 400 μg folic acid, 5·2 μg vitamin B12, 100 mg vitamin C, 45 μg vitamin K, 20 mg iron, 15 mg zinc, 4 mg copper, 280 mg calcium, 190 mg phosphorus, 200 mg potassium, 65 mg magnesium, 130 μg selenium, 250 μg iodine, and 2·6 mg manganese, provided daily. A second daily lipid-based protein-energy supplement was given if they had a BMI <20 kg/m^2^ at any time while receiving the original supplement or had weight gain in the second or third trimesters of pregnancy less than the Institute of Medicine's guidelines; if consumed completely, this additional supplement provided around 300 kcal and 11 g protein	No nutrient supplement provided by the study	12–14 weeks' gestation†	Ultrasound in Guatemala, India, and Pakistan; no plausible gestational age determination in DR Congo

The UNIMMAP formulation consisted of 800 μg per day vitamin A, 5 μg per day vitamin D, 10 mg per day vitamin E, 70 mg per day vitamin C, 1·4 mg per day vitamin B1, 1·4 mg per day vitamin B2, 18 mg per day niacin, 1·9 mg per day vitamin B6, 2·6 μg per day vitamin B12, 400 μg per day folic acid, 30 mg per day iron, 15 mg per day zinc, 2 mg per day copper, 65 μg per day selenium, and 150 μg per day iodine. SQ-LNS formulations were provided as 20 g sachets. LMP=last menstrual period. MMS=multiple micronutrient supplementation. RDA=recommended dietary allowance. RE=retinol equivalents. SQ-LNS=small-quantity lipid-based nutrient supplementation. UNIMMAP=United Nations International Multiple Micronutrient Antenatal Preparation.

* 60 mg iron and 400 μg folic acid.

† The study group that started supplementation from the preconceptional period was excluded, because the analysis focused on the effect of prenatal supplementation initiated during pregnancy.

**Table 2:** Ten-group and four-group categorisation of newborn types for the analysis of MMS and SQ-LNS

	**MMS analysis (14 studies)**			**SQ-LNS analysis (4 studies)**		
	Total (n=42 618)	Control (n=23 292)	MMS (n=19 326)	Total (n=6246)	Control (n=3832)	SQ-LNS (n=2414)
**Ten-group categorisation**						
Preterm–SGA–LBW	1046 (2·5%)	623 (2·7%)	423 (2·2%)	149 (2·4%)	100 (2·6%)	49 (2·0%)
Preterm–AGA–LBW	1863 (4·4%)	1055 (4·5%)	808 (4·2%)	331 (5·3%)	225 (5·9%)	106 (4·4%)
Preterm–LGA–LBW	170 (0·4%)	104 (0·5%)	66 (0·3%)	65 (1·0%)	41 (1·1%)	24 (1·0%)
Term–SGA–LBW	7996 (18·8%)	4503 (19·3%)	3493 (18·1%)	1236 (19·8%)	853 (22·3%)	383 (15·9%)
Term–AGA–LBW	147 (0·3%)	78 (0·3%)	69 (0·4%)	34 (0·5%)	23 (0·6%)	11 (0·5%)
Preterm–AGA–non-LBW	1984 (4·7%)	1096 (4·7%)	888 (4·6%)	333 (5·3%)	205 (5·4%)	128 (5·3%)
Preterm–LGA–non-LBW	1735 (4·1%)	859 (3·7%)	876 (4·5%)	251 (4·0%)	154 (4·0%)	97 (4·0%)
Term–SGA–non-LBW	8480 (19·9%)	4652 (20·0%)	3828 (19·8%)	1260 (20·2%)	815 (21·3%)	445 (18·4%)
Term–AGA–non-LBW	18 244 (42·8%)	9839 (42·2%)	8405 (43·5%)	2493 (39·9%)	1378 (36·0%)	1115 (46·2%)
Term–LGA–non-LBW	953 (2·2%)	483 (2·1%)	470 (2·4%)	94 (1·5%)	38 (1·0%)	56 (2·3%)
**Four-group categorisation**						
Term–non-SGA	19 344 (45·4%)	10 400 (44·7%)	8944 (46·3%)	2621 (42·0%)	1439 (37·6%)	1182 (49·0%)
Term–SGA	16 476 (38·7%)	9155 (39·3%)	7321 (37·9%)	2496 (40·0%)	1668 (43·5%)	828 (34·3%)
Preterm–non-SGA	5752 (13·5%)	3114 (13·4%)	2638 (13·7%)	980 (15·7%)	625 (16·3%)	355 (14·7%)
Preterm–SGA	1046 (2·5%)	623 (2·7%)	423 (2·2%)	149 (2·4%)	100 (2·6%)	49 (2·0%)

Data are n (%). Percentages might not sum to 100 due to rounding. Preterm birth is defined as gestational age at birth less than 37 completed weeks. Term birth is defined as gestational age at birth of 37 completed weeks or above. SGA is defined as birthweight less than the 10th percentile for sex and gestational age based on the INTERGROWTH-21st newborn size standards. AGA is defined as birthweight between 10th and 90th percentiles for sex and gestational age based on the INTERGROWTH-21^st^ newborn size standards. LGA is defined as birthweight larger than the 90th percentile for sex and gestational age based on the INTERGROWTH-21^st^ newborn size standards. Non-LBW is defined as a birthweight of 2500 g or greater. LBW is defined as a birthweight less than 2500 g. AGA=appropriate for gestational age. LBW=low birthweight. LGA=large for gestational age. MMS=multiple micronutrient supplementation. SGA=small for gestational age. SQ-LNS=small-quantity lipid-based nutrient supplementation.

Based on the random-effects models (table 3), compared with term**–**AGA**–**non-LBW, prenatal MMS led to a 27% lower risk of giving birth to a neonate who is preterm**–**SGA**–**LBW (RR 0·73, 95% CI 0·64–0·84; p=0·0003); an 18% lower risk of preterm**–**AGA**–**LBW (0·82, 0·74–0·91; p=0·0010); an 11% lower risk of preterm**–**AGA**–**non-LBW (0·89, 0·80–0·98; p=0·019); a 9% lower risk of term**–**SGA**–**LBW (0·91, 0·85–0·96; p=0·0046); and a 5% lower risk of term**–**SGA**–**non-LBW (0·95, 0·90–1·00; p=0·050). Prenatal MMS also appeared to reduce the risk of giving birth to a preterm**–**LGA–LBW neonate (0·71, 0·49–1·03), although the random-effects estimate was not significant (p=0·065). Prenatal SQ-LNS led to a 22% lower risk of giving birth to a preterm–LGA–non-LBW neonate (0·78, 0·65–0·94; p=0·023).

**Table 3:** Effects of prenatal MMS and SQ-LNS on newborn types based on the ten-group categorisation

		**Term–AGA–non-LBW**	**Preterm–SGA–LBW**	**Preterm–AGA–LBW**	**Preterm–LGA–LBW**	**Term–SGA–LBW**	**Term–AGA–LBW**	**Preterm–AGA–non-LBW**	**Preterm–LGA–non-LBW**	**Term–SGA–non-LBW**	**Term–LGA–non-LBW**
**MMS**											
Studies											
	Christian et al (2003)*^29^	1 (ref)	0·66 (0·36–1·23)	0·63 (0·40–0·99)	1·08 (0·29–3·98)	0·96 (0·83–1·10)	0·51 (0·12–2·13)	0·93 (0·66–1·33)	1·66 (1·04–2·65)	1·05 (0·89–1·23)	2·08 (0·51–8·42)
	Ramakrishnan et al (2003)^30^	1 (ref)	0·47 (0·04–5·16)	1·40 (0·40–4·88)	NA†	0·90 (0·48–1·66)	NA‡	1·87 (0·17–20·44)	NA†	0·82 (0·61–1·10)	0·48 (0·15–1·56)
	Friis et al (2004)^31^	1 (ref)	0·69 (0·16–3·05)	1·03 (0·53–1·98)	0·46 (0·04–5·03)	0·65 (0·40–1·07)	NA§	0·82 (0·51–1·32)	0·73 (0·46–1·17)	0·92 (0·68–1·23)	1·52 (0·71–3·28)
	Osrin et al (2005)^32^	1 (ref)	1·00 (0·47–2·12)	0·60 (0·30–1·20)	NA‡	0·68 (0·52–0·90)	1·70 (0·16–18·68)	0·86 (0·33–2·25)	0·86 (0·05–13·60)	0·89 (0·73–1·08)	1·69 (0·43–6·70)
	Fawzi et al (2007)^33^	1 (ref)	0·93 (0·58–1·48)	0·85 (0·65–1·10)	0·88 (0·50–1·55)	0·74 (0·59–0·92)	0·96 (0·06–15·26)	1·01 (0·84–1·20)	1·03 (0·88–1·20)	0·82 (0·73–0·92)	0·94 (0·78–1·14)
	Zeng et al (2008)*^34^	1 (ref)	0·41 (0·12–1·40)	0·94 (0·47–1·89)	0·36 (0·05–2·91)	0·96 (0·65–1·42)	NA§	0·90 (0·57–1·42)	1·33 (0·81–2·18)	0·98 (0·83–1·15)	1·22 (0·95–1·58)
	Roberfroid et al (2008)^35^	1 (ref)	1·00 (0·38–2·63)	1·08 (0·63–1·86)	0·67 (0·19–2·35)	0·98 (0·69–1·40)	NA§	1·00 (0·56–1·79)	1·70 (0·85–3·39)	0·94 (0·72–1·22)	2·30 (0·60–8·82)
	Bhutta et al (2009)*^36^	1 (ref)	0·61 (0·22–1·69)	1·02 (0·64–1·61)	0·76 (0·40–1·44)	1·00 (0·75–1·34)	NA¶	0·85 (0·57–1·27)	1·00 (0·82–1·22)	0·96 (0·74–1·25)	1·44 (0·88–2·37)
	Persson et al (2012)^37^	1 (ref)	0·76 (0·45–1·26)	0·78 (0·46–1·32)	NA‡	0·99 (0·88–1·10)	0·25 (0·03–2·01)	1·16 (0·46–2·92)	NA‡	1·01 (0·93–1·10)	0·50 (0·06–4·46)
	Moore et al (2012)^38^	1 (ref)	NA†	0·93 (0·19–4·56)	NA‡	0·83 (0·51–1·35)	NA†	NA†	0·47 (0·04–5·12)	0·98 (0·76–1·28)	1·82 (0·63–5·25)
	West et al (2014)*^39^	1 (ref)	0·69 (0·60–0·80)	0·80 (0·71–0·90)	0·50 (0·25–0·99)	0·91 (0·87–0·94)	1·15 (0·78–1·68)	0·82 (0·72–0·95)	1·02 (0·82–1·28)	0·97 (0·93–1·02)	1·55 (0·80–3·02)
	Ashorn et al (2015)^40^	1 (ref)	0·47 (0·12–1·87)	0·86 (0·39–1·92)	NA†	1·03 (0·68–1·57)	NA†	0·66 (0·26–1·71)	0·93 (0·24–3·69)	0·74 (0·55–0·99)	0·81 (0·38–1·71)
	Adu-Afarwuah et al (2015)^41^	1 (ref)	1·38 (0·40–4·85)	0·52 (0·19–1·38)	0·93 (0·06–14·78)	0·65 (0·39–1·08)	NA†	0·79 (0·36–1·74)	NA‖	0·93 (0·54–1·61)	1·45 (0·82–2·55)
	Bliznashka et al (2022)*^42^	1 (ref)	2·30 (0·77–6·90)	0·95 (0·54–1·65)	0·64 (0·20–1·99)	0·93 (0·58–1·48)	NA†	0·87 (0·67–1·12)	1·19 (1·00–1·41)	1·13 (0·75–1·70)	1·57 (0·69–3·56)
Pooled, fixed-effect		1 (ref)	0·73 (0·65–0·82)	0·82 (0·75–0·90)	0·71 (0·52–0·97)	0·91 (0·88–0·94)	1·05 (0·74–1·50)	0·89 (0·81–0·97)	1·08 (0·99–1·18)	0·96 (0·93–0·99)	1·12 (0·99–1·27)
Pooled, random-effects		1 (ref)	0·73 (0·64–0·84)	0·82 (0·74–0·91)	0·71 (0·49–1·03)	0·91 (0·85–0·96)	1·05 (0·64–1·74)	0·89 (0·80–0·98)	1·08 (0·97–1·21)	0·95 (0·90–1·00)	1·15 (0·98–1·36)
Heterogeneity											
	*I*^2^	NA	0	0	0	13·43%	0	0	10·58%	14·88%	6·88%
	p value	NA	0·67	0·93	0·96	0·31	0·53	0·97	0·34	0·29	0·38
**SQ-LNS**											
Studies											
	Ashorn et al (2015)^40^	1 (ref)	0·79 (0·24–2·56)	1·02 (0·48–2·20)	NA‡	1·04 (0·69–1·58)	NA†	0·48 (0·17–1·39)	0·48 (0·09–2·58)	0·83 (0·63–1·11)	1·01 (0·50–2·05)
	Adu-Afarwuah et al (2015)^41^	1 (ref)	0·96 (0·24–3·80)	0·27 (0·08–0·96)	NA†	0·35 (0·18–0·67)	NA‡	1·24 (0·62–2·50)	1·12 (0·38–3·28)	0·96 (0·56–1·66)	1·63 (0·94–2·84)
	Matias et al (2016)*^43^	1 (ref)	0·90 (0·58–1·37)	0·82 (0·57–1·17)	0·63 (0·30–1·33)	0·92 (0·82–1·03)	0·50 (0·20–1·26)	0·89 (0·67–1·19)	0·88 (0·60–1·30)	0·96 (0·88–1·04)	1·48 (0·39–5·66)
	Hambidge et al (2019)**^44^	1 (ref)	0·65 (0·42–1·01)	0·75 (0·55–1·03)	1·19 (0·78–1·81)	0·76 (0·68–0·86)	1·68 (0·64–4·40)	0·86 (0·67–1·10)	0·77 (0·68–0·87)	0·99 (0·91–1·08)	5·02 (1·19–21·12)
Pooled, fixed-effect		1 (ref)	0·78 (0·58–1·04)	0·77 (0·62–0·96)	1·02 (0·71–1·47)	0·84 (0·77–0·91)	0·90 (0·46–1·74)	0·88 (0·73–1·05)	0·78 (0·70–0·87)	0·97 (0·91–1·03)	1·51 (1·02–2·26)
Pooled, random-effects		1 (ref)	0·78 (0·48–1·25)	0·77 (0·52–1·15)	0·94 (0·02–47·39)	0·81 (0·48–1·36)	0·91 (0·00–2007·18)	0·88 (0·66–1·18)	0·78 (0·65–0·94)	0·97 (0·88–1·06)	1·55 (0·67–3·60)
Heterogeneity											
	*I*^2^	NA	0	8·07%	53·17%	76·56%	68·36%	0	0	0	24·78%
	p value	NA	0·77	0·35	0·14	0·0051	0·075	0·53	0·75	0·71	0·26

Data are risk ratios (95% CI), unless otherwise specified. Risk ratios and 95% CIs were computed using log-binomial or modified Poisson models. Estimates are not available for models with 0 events or with few events due to failure of model convergence. AGA=appropriate for gestational age. LBW=low birthweight. LGA=large for gestational age. MMS=multiple micronutrient supplementation. NA=not applicable. SGA=small for gestational age. SQ-LNS=small-quantity lipid-based nutrient supplementation.

* Poisson models with cluster-robust standard errors were used.

† Only one event.

‡ Zero events.

§ Only three events.

¶ Only two events.

‖ Only six events.

** The study arm that started supplementation from the preconceptional period was excluded as the analysis focused on the effect of prenatal supplementation initiated during pregnancy; Poisson models with cluster-robust standard errors were used to account for country.

For the four-group categorisation, in the random-effects models, prenatal MMS led to a 29% lower risk of giving birth to a preterm–SGA neonate (0·71, 0·62–0·82; p=0·0002) and a 7% lower risk of term–SGA (0·93, 0·89–0·98; p=0·0066), compared with term–non-SGA (table 4). Prenatal MMS also appeared to reduce the risk of preterm–non-SGA (0·93, 0·87–1·00), although the effect was not significant (p=0·061). In the random-effects models, prenatal SQ-LNS did not significantly affect the risk of the small vulnerable newborn types based on the four-group categorisation (table 4). No publication bias was detected on the basis of the funnel plots (appendix pp 19–29).

**Table 4:** Effects of prenatal MMS and SQ-LNS on newborn types based on the four-group categorisation

		**Term–non-SGA**	**Term–SGA**	**Preterm–non-SGA**	**Preterm–SGA**
**MMS**					
Christian et al (2003)*^29^		1 (ref)	1·00 (0·91–1·09)	0·95 (0·77–1·17)	0·66 (0·36–1·23)
Ramakrishnan et al (2003)^30^		1 (ref)	0·86 (0·67–1·11)	1·87 (0·65–5·40)	0·48 (0·04–5·26)
Friis et al (2004)^31^		1 (ref)	0·84 (0·66–1·06)	0·82 (0·62–1·08)	0·67 (0·15–2·97)
Osrin et al (2005)^32^		1 (ref)	0·84 (0·73–0·97)	0·69 (0·40–1·17)	0·99 (0·46–2·09)
Fawzi et al (2007)^33^		1 (ref)	0·82 (0·74–0·90)	0·99 (0·90–1·09)	0·93 (0·58–1·49)
Zeng et al (2008)*^34^		1 (ref)	0·97 (0·83–1·12)	0·99 (0·73–1·35)	0·40 (0·12–1·38)
Roberfroid et al (2008)^35^		1 (ref)	0·96 (0·79–1·17)	1·11 (0·82–1·52)	1·00 (0·38–2·62)
Bhutta et al (2009)*^36^		1 (ref)	0·96 (0·79–1·16)	0·94 (0·82–1·08)	0·58 (0·21–1·64)
Persson et al (2012)^37^		1 (ref)	1·01 (0·95–1·06)	0·87 (0·55–1·36)	0·77 (0·46–1·28)
Moore et al (2012)^38^		1 (ref)	0·94 (0·76–1·17)	0·62 (0·18–2·16)	Low events (n=1)
West et al (2014)*^39^		1 (ref)	0·95 (0·93–0·98)	0·86 (0·79–0·93)	0·69 (0·60–0·80)
Ashorn et al (2015)^40^		1 (ref)	0·85 (0·68–1·07)	0·84 (0·49–1·44)	0·48 (0·12–1·88)
Adu-Afarwuah et al (2015)^41^		1 (ref)	0·76 (0·53–1·09)	0·55 (0·31–0·96)	1·34 (0·38–4·69)
Bliznashka et al (2022)*^42^		1 (ref)	1·02 (0·71–1·48)	1·02 (0·91–1·16)	Low events (n=9)
Pooled, fixed-effect		1 (ref)	0·95 (0·93–0·97)	0·93 (0·89–0·97)	0·71 (0·63–0·81)
Pooled, random-effects		1 (ref)	0·93 (0·89–0·98)	0·93 (0·87–1·00)	0·71 (0·62–0·82)
**Heterogeneity**					
	*I*^2^	NA	38·16%	25·59%	0
	p value	NA	0·073	0·18	0·92
**SQ-LNS**					
	Ashorn et al (2015)^40^	1 (ref)	0·91 (0·73–1·13)	0·74 (0·42–1·30)	0·79 (0·24–2·55)
	Adu-Afarwuah et al (2015)^41^	1 (ref)	0·61 (0·41–0·90)	0·82 (0·50–1·33)	0·92 (0·23–3·64)
	Matias et al (2016)*^43^	1 (ref)	0·96 (0·91–1·02)	0·88 (0·73–1·07)	0·91 (0·59–1·39)
	Hambidge et al (2019)†^44^	1 (ref)	0·89 (0·87–0·90)	0·85 (0·73–0·99)	0·64 (0·41–1·00)
Pooled, fixed-effect		1 (ref)	0·89 (0·88–0·91)	0·85 (0·76–0·96)	0·77 (0·58–1·04)
Pooled, random-effects		1 (ref)	0·90 (0·77–1·06)	0·85 (0·71–1·03)	0·77 (0·48–1·25)
**Heterogeneity**					
	*I*^2^	NA	72·41%	0	0
	p value	NA	0·012	0·94	0·73

Data are risk ratio (95% CI), unless otherwise specified. Risk ratios and 95% CIs were computed using log-binomial or modified Poisson models. Estimates are not available for models marked with few events due to failure of model convergence. MMS=multiple micronutrient supplementation. NA=not applicable. SGA=small for gestational age. SQ-LNS=small-quantity lipid-based nutrient supplementation.

* Poisson models with cluster-robust standard errors were used.

† The study arm that started supplementation from the preconceptional period was excluded as the analysis focused on the effect of prenatal supplementation initiated during pregnancy; Poisson models with cluster-robust standard errors were used to account for country.

In exploratory subgroup analyses (appendix p 11), the protective effect of MMS on the three small vulnerable newborn types was stronger among nulliparous participants than among multiparous participants (p for interaction=0·024 for term–SGA, p for interaction=0·044 for preterm–non-SGA, and p for interaction=0·042 for preterm–SGA). A protective effect of SQ-LNS on preterm–non-SGA was observed among multiparous participants and not nulliparous participants (p for interaction=0·0010). A protective effect of SQ-LNS against term–SGA was observed among participants enrolled before 20 weeks of gestation but not those enrolled at 20 weeks of gestation or later (p for interaction=0·023). The effect of SQ-LNS on term–SGA varied by maternal early-pregnancy BMI status but remained protective in all three BMI strata including underweight, normal weight, and overweight and obesity (p for interaction <0·0001). The effects of SQ-LNS on term–SGA and preterm–non-SGA were stronger among participants with mild anaemia and moderate to severe anaemia than among those without anaemia (p for interaction <0·0001 for both newborn types).

When adjusting for maternal age, parity, gestational age at study enrolment, maternal early-pregnancy BMI, and maternal haemoglobin concentrations at baseline, the resultant pooled estimates were similar to those from the primary analyses (appendix pp 12–13). In the sensitivity analysis using studies with ultrasound-based measures of gestation age, six studies (n=5526) were included for the MMS analysis and three studies (n=1924) for the SQ-LNS analysis. In the random-effects models, the point estimates for all small vulnerable newborn types were congruent with those from the primary analysis (appendix pp 14–15). However, all small vulnerable newborn types did not retain significance, except for the effect of MMS on term–SGA. In the secondary analysis on neonatal mortality (appendix p 16), no significant effects were found for prenatal MMS (RR 1·10, 95% CI 0·95–1·28) or prenatal SQ-LNS (1·06, 0·49–2·31) on neonatal mortality.

Of the 16 studies included in this analysis, five were assessed to have a low risk of bias, and 11 were assessed to have some concerns due to missing outcome data or outcome measurement using the last menstrual period. No studies were determined to have a high risk of bias (appendix p 17).

## Discussion

In this individual participant data meta-analysis in LMICs, we showed that prenatal MMS significantly reduced the risk of five of nine small vulnerable newborn types in the ten-group categorisation and of term–SGA and preterm–SGA in the four-group categorisation. Prenatal SQ-LNS reduced the risk of preterm–LGA–non-LBW in the ten-group categorisation, but no significant effects were found on small vulnerable newborns in the four-group categorisation.

Prenatal MMS had the greatest protective effects against the risk of giving birth to a neonate who is preterm–SGA–LBW (a 27% reduction in risk) or preterm–AGA–LBW (an 18% reduction in risk). Prenatal MMS might also reduce the risk of giving birth to a preterm–LGA–LBW neonate, although the random-effects estimate was not significant, which could be attributed to the rarity of this type (less than 0·50% in the analytical sample for the MMS analysis). An analysis of population-based birth cohorts in LMICs showed that preterm–LGA–LBW, preterm–AGA–LBW, and preterm–SGA–LBW were the three subtypes with the greatest mortality risk compared with term–AGA–non-LBW.^7^ Therefore, this study demonstrates the benefits of prenatal MMS in preventing the small vulnerable newborn types with the greatest mortality risks. Similarly, in the four-group categorisation, prenatal MMS resulted in a 29% reduction in the risk of giving birth to a preterm–SGA neonate, the type associated with the highest risk of neonatal mortality (RR 10·4, 95% CI 8·5–14·5).^7^ The benefits of prenatal MMS on preterm–non-SGA and term–SGA were modest (7% risk reduction for both types). However, preterm–non-SGA and term–SGA are much more common than preterm–SGA.^6^ These two subtypes are also associated with considerable neonatal mortality (preterm–non-SGA 6·0, 4·1–14·5; term–SGA 2·7, 2·1–4·0),^7^ and jointly explain almost half (47·5%) of all neonatal mortality globally.^3^ Therefore, the benefits of prenatal MMS in reducing the long-term adverse consequences of these small vulnerable newborns are expected to be substantial. Accumulating evidence supports that prenatal MMS reduces the risks of giving birth to a neonate who is SGA–LBW and might also prevent preterm birth compared with iron and folic acid alone.^5^, ^10^, ^11^ There are increasing calls for using MMS as the standard of antenatal care.^45^, ^46^ The protective effects of prenatal MMS on the risk of numerous small vulnerable newborn types, especially the types with the greatest mortality risk, lend additional support for the switch to MMS as the standard antenatal care.

We showed that prenatal SQ-LNS had a significant protective effect against giving birth to a preterm–LGA–non-LBW neonate (a 22% reduction in risk) but not the other small vulnerable newborns. The overall absence of significant effects for SQ-LNS might be related to the small number of SQ-LNS studies included. The Hartung–Knapp–Sidik–Jonkman method used to adjust for the small number of studies in the random-effects models has led to conservative, wide CIs. Furthermore, we focused on the effects of SQ-LNS that provided less than 120 kcal per day of energy.^17^ The effect of prenatal LNSs with greater energy content is an active area of research.

The subgroup analyses were exploratory and underpowered. They showed that the effects of SQ-LNS on small vulnerable newborns might be stronger among participants initiating supplementation before 20 weeks of gestation, a finding in line with previous evidence that underscores the importance of initiating nutritional supplements from early pregnancy.^10^ The exploratory subgroup analyses also showed evidence of potential modification of the effects of MMS and SQ-LNS by parity and the effect of SQ-LNS by maternal early-pregnancy BMI and anaemia status. These potential effect modifiers warrant further examination in future studies.

The protective effects of MMS and SQ-LNS on small vulnerable newborn types contradict, to some extent, systematic reviews that found no substantial benefits of MMS or SQ-LNS on neonatal mortality, a finding we also observed in our analytical sample.^11^, ^15^ The absence of significant effects on neonatal mortality in the literature and this study might be due to the rarity of the small vulnerable newborn types that confer the greatest mortality risk, which might have masked the effect of the nutritional supplements on neonatal mortality in the overall sample. The three small vulnerable newborn types with the greatest mortality risk (ie, preterm–LGA–LBW, preterm–AGA–LBW, and preterm–SGA–LBW)^7^ combined account for less than 10% of the analytical sample in this study. As small vulnerable newborns also have elevated risks of growth faltering, non-communicable diseases, and reduced learning potential in later life, the benefits of prenatal nutritional supplements on reducing newborn vulnerability extend beyond the short-term prevention of neonatal mortality to long-term gains in human capital.^3^

This study has several limitations. First, sparse-data bias is an important limitation that should be highlighted. As newborns were partitioned into small vulnerable newborn types, the number of events for several comparisons became small. Meta-analysis remains an important approach to investigating intervention effects on rare outcomes. Future studies powered specifically for small vulnerable newborn types are needed. Second, studies that relied on the last menstrual period for gestational age estimation might have measurement errors for SGA–preterm birth outcomes. In the sensitivity analysis restricted to studies using ultrasound, the estimates for almost all newborn types did not have statistical significance. The point estimates were similar to those from the primary analyses, suggesting that the lack of statistical significance might be due to the reduced statistical power. Third, we did not adjust for multiple comparisons, which might have increased the probability of type 1 errors for the composite null hypothesis that none of the small vulnerable newborn types were affected by the nutritional supplements. However, quantitative adjustments for multiple testing would have inflated the probability of type 2 error and masked potentially important effects.^24^ Fourth, the analysis of SQ-LNS included only four studies, which might lead to inaccurate variance estimates in the pooled results. Fifth, the percentage of participants with missing outcome data was high.

In conclusion, prenatal MMS and SQ-LNS reduce the risk of small vulnerable newborn types to varying extents. The protective effects are particularly substantial for small vulnerable newborn types conferring the greatest risk of neonatal mortality. This work underscores the importance of nutritional supplements in antenatal care in LMICs. Efforts are also needed to address the socioeconomic drivers of poor birth outcomes, such as poverty, poor maternal education, low access to quality antenatal care, and maternal and child food insecurity.

### Contributors

### Data sharing

Access to all individual-level data that comprise the pooled dataset used in this study is restricted to approved individuals at the Bill & Melinda Gates Foundation and the Harvard TH Chan School of Public Health based on terms set forth in their Data Use Agreements. Reasonable requests from qualified researchers will be considered for data sharing. These requests should be submitted to ghp@hsph.harvard.edu.

## Declaration of interests

We declare no competing interests.
