# Supplementary material for: The effects of prenatal multiple micronutrient supplementation and small-quantity lipid-based nutrient supplementation on small vulnerable newborn types in low-income and middle-income countries: a meta-analysis of individual participant data
Source: Lancet Glob Health. 2025 Jan 29;13(2):e298–308. doi: 10.1016/S2214-109X(24)00449-2 (PMC11782989; doi:10.1016/S2214-109X(24)00449-2)
Supplement: Supplementary appendix [file mmc1.pdf]

THE LANCET
Global Health

Supplementary appendix

This appendix formed part of the original submission and has been peer reviewed. We post it as supplied by the authors.

Supplement to: Wang D, Liu E, Perumal N, et al. The effects of prenatal multiple micronutrient supplementation and small-quantity lipid-based nutrient supplementation on small vulnerable newborn types in low-income and middle-income countries: a meta-analysis of individual participant data. *Lancet Glob Health* 2025; **13:** e298–308.

SUPPLEMENTARY MATERIALS

Table of Contents

Members of the Gestational Weight Gain Pooling Project Consortium 3

Supplemental Table 1. PubMed search strategy for identifying randomized controlled trials of nutrient supplements among pregnant women in low- and middle-income countries 8

Supplemental Table 2. Characteristics of the eligible studies not included in the individual participant data meta-analysis 9

Supplemental Table 3. Missingness in outcome data in the included trials on multiple micronutrient supplements and small-quantity lipid-based nutrient supplements 10

Supplemental Table 4. Effects of prenatal multiple micronutrient supplements and small-quantity lipid-based nutrient supplements on newborn types based on the four-group categorization by potential effect modifiers 11

Supplemental Table 5. Effects of prenatal multiple micronutrient supplements and small-quantity lipid-based nutrient supplements on newborn types based on the ten-group categorization, after adjusting for covariates when estimating the study-specific estimates 12

Supplemental Table 6. Effects of prenatal multiple micronutrient supplements and small-quantity lipid-based nutrient supplements on newborn types based on the four-group categorization, after adjusting for covariates when estimating the study-specific estimates 13

Supplemental Table 7. Effects of prenatal multiple micronutrient supplements and small-quantity lipid-based nutrient supplements on newborn types based on the ten-group categorization when restricting to studies with ultrasound-based measures of gestational age 14

Supplemental Table 8. Effects of prenatal multiple micronutrient supplements and small-quantity lipid-based nutrient supplements on newborn types based on the four-group categorization when restricting to studies with ultrasound-based measures of gestational age 15

Supplemental Table 9. Effects of prenatal multiple micronutrient supplements and small-quantity lipid-based nutrient supplements on neonatal mortality 16

Supplemental Table 10. Risk of bias of the included studies 17

Supplemental Figure 1. PRISMA flow diagram for the individual participant data meta-analysis on the effects of prenatal nutritional supplements on small vulnerable newborn types in low- and middle-income countries 18

Supplemental Figure 2. Funnel plot for the effect of prenatal multiple micronutrient supplements on the small vulnerable newborn type of term-SGA-nonLBW 19

Supplemental Figure 3. Funnel plot for the effect of prenatal multiple micronutrient supplements on the small vulnerable newborn type of term-SGA-LBW 19

Supplemental Figure 4. Funnel plot for the effect of prenatal multiple micronutrient supplements on the small vulnerable newborn type of preterm-SGA-LBW 20

Supplemental Figure 5. Funnel plot for the effect of prenatal multiple micronutrient supplements on the small vulnerable newborn type of preterm-AGA-nonLBW 20

Supplemental Figure 6. Funnel plot for the effect of prenatal multiple micronutrient supplements on the small vulnerable newborn type of preterm-AGA-LBW 21

Supplemental Figure 7. Funnel plot for the effect of prenatal multiple micronutrient supplements on the small vulnerable newborn type of term-AGA-LBW 21

Supplemental Figure 8. Funnel plot for the effect of prenatal multiple micronutrient supplements on the small vulnerable newborn type of term-LGA-nonLBW 22

Supplemental Figure 9. Funnel plot for the effect of prenatal multiple micronutrient supplements on the small vulnerable newborn type of preterm-LGA-nonLBW 22

Supplemental Figure 10. Funnel plot for the effect of prenatal multiple micronutrient supplements on the small vulnerable newborn type of preterm-LGA-LBW 23

Supplemental Figure 11. Funnel plot for the effect of prenatal small-quantity lipid-based nutrient supplements on the small vulnerable newborn type of term-SGA-nonLBW 23

Supplemental Figure 12. Funnel plot for the effect of prenatal small-quantity lipid-based nutrient supplements on the small vulnerable newborn type of term-SGA-LBW 24

Supplemental Figure 13. Funnel plot for the effect of prenatal small-quantity lipid-based nutrient supplements on the small vulnerable newborn type of preterm-SGA-LBW 24

Supplemental Figure 14. Funnel plot for the effect of prenatal small-quantity lipid-based nutrient supplements on the small vulnerable newborn type of preterm-AGA-nonLBW 25

Supplemental Figure 15. Funnel plot for the effect of prenatal small-quantity lipid-based nutrient supplements on the small vulnerable newborn type of preterm-AGA-LBW 25

Supplemental Figure 16. Funnel plot for the effect of prenatal small-quantity lipid-based nutrient supplements on the small vulnerable newborn type of term-LGA-nonLBW 26

Supplemental Figure 17. Funnel plot for the effect of prenatal small-quantity lipid-based nutrient supplements on the small vulnerable newborn type of preterm-LGA-nonLBW 26

Supplemental Figure 18. Funnel plot for the effect of prenatal multiple micronutrient supplements on the small vulnerable newborn type of term-SGA 27

Supplemental Figure 19. Funnel plot for the effect of prenatal multiple micronutrient supplements on the small vulnerable newborn type of preterm-nonSGA 27

Supplemental Figure 20. Funnel plot for the effect of prenatal multiple micronutrient supplements on the small vulnerable newborn type of preterm-SGA 28

Supplemental Figure 21. Funnel plot for the effect of prenatal small-quantity lipid-based nutrient supplements on the small vulnerable newborn type of term-SGA 28

Supplemental Figure 22. Funnel plot for the effect of prenatal small-quantity lipid-based nutrient supplements on the small vulnerable newborn type of preterm-nonSGA 29

Supplemental Figure 23. Funnel plot for the effect of prenatal small-quantity lipid-based nutrient supplements on the small vulnerable newborn type of preterm-SGA 29

References 30

Members of the Gestational Weight Gain Pooling Project Consortium

Seth Adu-Afarwuah: Department of Nutrition and Food Science, University of Ghana, Legon, Ghana

Per Ashorn: Center for Child, Adolescent and Maternal Health Research, Faculty of Medicine and Health Technology, Tampere University and Tampere University Hospital, Tampere, Finland

Ulla Ashorn: Center for Child, Adolescent and Maternal Health Research, Faculty of Medicine and Health Technology, Tampere University and Tampere University Hospital, Tampere, Finland

Malay Kanti Mridha: Center for Non-communicable Diseases and Nutrition, BRAC James P Grant School of Public Health, BRAC University, Dhaka, Bangladesh

Shams Arifeen: International Center for Diarrheal Disease Research, Bangladesh, Dhaka, Bangladesh

Zulfiqar A Bhutta: Centre for Global Child Health, Hospital for Sick Children and Institute for Global Health & Development, The Aga Khan University, Karachi, Pakistan

Yue Cheng: Department of Nutrition and Food Safety Research, School of Public Health, Xi'an Jiaotong University Health Science Center, Xi'an, Shaanxi, China

Parul Christian: Department of International Health, Bloomberg School of Public Health, Johns Hopkins University, Baltimore, Maryland, USA

Anthony M Costello: University College London Institute for Global Health, London, United Kingdom

Kathryn G Dewey: Department of Nutrition, University of California, Davis, Davis, California, USA

Henrik Friis: Department of Nutrition, Exercise and Sports, University of Copenhagen, Copenhagen, Denmark

Exnevia Gomo: Faculty of Medicine and Health Sciences, University of Zimbabwe, Harare, Zimbabwe

Rebecca Grais: Epicentre, Paris, France

Ousmane Guindo: Epicentre Niger, Niamey, Niger

Nancy F Krebs: University of Colorado School of Medicine, Aurora, Colorado, USA

Lieven Huybregts: Department of Food Technology, Safety and Health, Ghent University, Ghent, Belgium; Poverty, Health and Nutrition Division, International Food Policy Research Institute, Washington, DC, USA

Sheila Isanaka: Epicentre, Paris, France; Departments of Nutrition and Global Health and Population, Harvard TH Chan School of Public Health, Harvard University, Boston, Massachusetts, USA

Carl Lachat: Department of Food Technology, Safety and Health, Ghent University, Ghent, Belgium

Anna Lartey: Department of Nutrition and Food Science, University of Ghana, Legon, Ghana

Abu Ahmed Shamim: Center for Non-communicable Diseases and Nutrition, BRAC James P Grant School of Public Health, BRAC University, Dhaka, Bangladesh

Steven C LeClerq: Center for Human Nutrition, Department of International Health, Bloomberg School of Public Health, Johns Hopkins University, Baltimore, Maryland, USA

Kenneth Maleta: School of Public Health and Family Medicine, College of Medicine, University of Malawi, Blantyre, Malawi

Dharma S Manandhar: Mother and Infant Research Activities, Kathmandu, Nepal

Reynaldo Martorell: Hubert Department of Global Health, Rollins School of Public Health, Emory University, Atlanta, Georgia, USA

Susana L Matias: Department of Nutritional Sciences and Toxicology, University of California, Berkeley, California, USA

Elizabeth M McClure: RTI International, Durham, North Carolina, USA

Sophie E Moore: Department of Women and Children's Health, King's College London, London, United Kingdom; St Thomas' Hospital, London; MRC Unit The Gambia at London School of Hygiene & Tropical Medicine, Banjul, The Gambia

David Osrin: University College London Institute for Global Health, London, United Kingdom

Willy Urassa: Department of Microbiology and Immunology, Muhimbili University of Health and Allied Sciences, Dar es Salaam, Tanzania

Andrea B Pembe: Department of Obstetrics and Gynaecology, Muhimbili University of Health and Allied Sciences, Dar es Salaam, Tanzania

Andrew M Prentice: MRC Unit The Gambia at London School of Hygiene & Tropical Medicine, Banjul, The Gambia

Usha Ramakrishnan: Hubert Department of Global Health, Rollins School of Public Health, Emory University, Atlanta, Georgia, USA

Juan Rivera: National Institute of Public Health, Mexico, Cuernavaca, Morelos, Mexico

Arjumand Rizvi: Center of Excellence in Women and Child Health, The Aga Khan University, Karachi, Pakistan

Dominique Roberfroid: Namur University, Namur, Belgium; Belgian Health Care Knowledge Centre, Brussels, Belgium

Sajid Soofi: Center of Excellence in Women and Child Health, The Aga Khan University, Karachi, Pakistan

Kerry Schulze: Center for Human Nutrition, Department of International Health, Bloomberg School of Public Health, Johns Hopkins University, Baltimore, Maryland, USA

Keith P West Jr: Center for Human Nutrition, Department of International Health, Bloomberg School of Public Health, Johns Hopkins University, Baltimore, Maryland, USA

Lee Wu: Center for Human Nutrition, Department of International Health, Bloomberg School of Public Health, Johns Hopkins University, Baltimore, Maryland, USA

Lingxia Zeng: Department of Epidemiology and Biostatistics, School of Public Health, Xi'an Jiaotong University Health Science Center, Xi'an, Shaanxi, China

Zhonghai Zhu: Department of Epidemiology and Biostatistics, School of Public Health, Xi'an Jiaotong University Health Science Center, Xi'an, Shaanxi, China

Supplemental Table 1. PubMed search strategy for identifying randomized controlled trials of nutrient supplements among pregnant women in low- and middle-income countries

Concept	PubMed Search terms
(1) Clinical trials	(clinical[tiab] AND trial[tiab]) OR "clinical trials as topic"[mesh] OR "clinical trial"[pt] OR random*[tiab] OR "random allocation"[mesh] OR "therapeutic use"[sh] OR “controlled trial”[tiab] OR “interventional study”[tiab] OR “single-arm trial”[tiab] OR “clinical study”[tiab] OR “Phase II trial”[tiab] OR “Phase II study”[tiab] OR “Phase I/II trial”[tiab] OR “Phase I/II study”[tiab]
(2) Pregnancy	"Pregnancy"[Mesh] OR Pregnanc*[tiab] OR Pregnant[tiab] OR prenatal[tiab] or gestation*[tiab] or antenatal[tiab]
(3) Nutrient supplements	Vitamins[Mesh] OR Micronutrients[Mesh] OR Dietary Supplements[Mesh] OR calcium[tiab] OR magnesium[tiab] OR phosphorus[tiab] OR potassium[tiab] OR boron[tiab] OR cobalt[tiab] OR chromium[tiab] OR copper[tiab] OR iodine[tiab] OR iron[tiab] OR manganese[tiab] OR molybdenum[tiab] OR selenium[tiab] OR zinc[tiab] OR vitamin*[tiab] OR thiamin*[tiab] or riboflavin[tiab] or niacin[tiab] OR “pantothenic acid”[tiab] OR pyridox*[tiab] OR biotin[tiab] OR folate[tiab] OR “folic acid”[tiab] OR cobalamin*[tiab] OR retinol[tiab] OR “ascorbic acid”[tiab] OR ergocalciferol[tiab] OR cholecaliferol[tiab] OR tocopherol*[tiab] OR tocotrienol*[tiab] OR phylloquinone[tiab] OR menaquinone[tiab] OR carotenoid*[tiab] or carotene[tiab] OR antioxidant*[tiab] OR “Balanced protein energy supplements”[tiab] OR “Lipid-based nutrient supplements”[tiab] OR “LNS”[tiab]
(4) Low- and middle-income countries	("Developing Countries"[mesh] OR developing countr*[tiab] OR developing nation*[tiab] OR less developed countr*[tiab] OR less developed nation*[tiab] OR third world nation*[tiab] OR third world countr*[tiab] OR under developed nation*[tiab] OR underdeveloped nation*[tiab] OR under developed countr*[tiab] OR underdeveloped nation*[tiab] OR middle income countr*[tiab] OR middle income nation*[tiab] OR low income countr*[tiab] OR low income nation*[tiab] OR poor countr*[tiab] OR poor nation*[tiab] OR lmic[tiab] OR lmics[tiab] OR "Africa"[mesh] OR "Asia"[mesh] OR "South America"[mesh] OR "Latin America"[mesh] OR "Central America"[mesh] OR africa[tiab] OR asia[tiab] OR south america*[tiab] OR latin america*[tiab] OR central america*[tiab] OR Afghanistan*[tiab] OR Albania*[tiab] OR Algeria*[tiab] OR Samoa*[tiab] OR Angola*[tiab] OR Armenia*[tiab] OR Azerbaijan*[tiab] OR Bangladesh*[tiab] OR Bengali[tiab] OR Belarus*[tiab] OR Belize[tiab] OR Benin[tiab] OR Bhutan*[tiab] OR Bolivia*[tiab] OR Bosnia*[tiab] OR Herzegovina*[tiab] OR Botswana*[tiab] OR Brazil*[tiab] OR Bulgaria*[tiab] OR Burkina Faso[tiab] OR Burundi*[tiab] OR Cabo Verd*[tiab] OR Cape Verd*[tiab] OR Cambodia*[tiab] OR Cameroon*[tiab] OR Central African*[tiab] OR Chad*[tiab] OR China[tiab] OR Chinese[tiab] OR Colombia*[tiab] OR Comoros[tiab] OR Congo[tiab] OR Cook Islands[tiab] OR Costa Rica*[tiab] OR Cote d'Ivoire[tiab] OR Ivory Coast[tiab] OR Cuba[tiab] OR Cuban[tiab] OR Djibouti[tiab] OR Dominica* [tiab] OR Ecuador[tiab] OR Egypt[tiab] OR El Salvador*[tiab] OR Eritrea*[tiab] OR Ethiopia*[tiab] OR Falkland Islands[tiab] OR Fiji*[tiab] OR Gabon*[tiab] OR Gambia*[tiab] OR Georgia*[tiab] OR Ghana*[tiab] OR Grenada*[tiab] OR Guadeloupe[tiab] OR Guatemala*[tiab] OR Guian*[tiab] OR Guinea*[tiab] OR Guyan*[tiab] OR Haiti*[tiab] OR Hondura*[tiab] OR India[tiab] OR Indian*[tiab] OR Indonesia*[tiab] OR Iran*[tiab] OR Iraq*[tiab] OR Jamaica*[tiab] OR Jordan*[tiab] OR Kazakh*[tiab] OR Kenya*[tiab] OR Kiribati[tiab] OR People's Republic of Korea[tiab] OR North Korea[tiab] OR Kosovo[tiab] OR Kosovar*[tiab] OR Kyrgyz*[tiab] OR Lao[tiab] OR Laos[tiab] OR Laotian*[tiab] OR Lebanon[tiab] OR Lebanes*[tiab] OR Lesotho[tiab] OR Liberia*[tiab] OR Libya*[tiab] OR Macedonia*[tiab] OR Madagascar*[tiab] OR Malawi*[tiab] OR Malvinas[tiab] OR Malaysia*[tiab] OR Maldives[tiab] OR Mali[tiab] OR Marshall Island*[tiab] OR Mauritania*[tiab] OR Mauriti*[tiab] OR Mayotte[tiab] OR Mexico[Mesh] OR Mexican*[tw] OR Micronesia*[tiab] OR Moldova*[tiab] OR Mongolia*[tiab] OR Montenegr*[tiab] OR Montserrat[tiab] OR Morocc*[tiab] OR Mozambique[tiab] OR Myanmar[tiab] OR Burmese*[tiab] OR Burma[tiab] OR Namibia*[tiab] OR Nauru[tiab] OR Nepal*[tiab] OR Netherlands Antilles[tiab] OR Nicaragua*[tiab] OR Niger*[tiab] OR Niue[tiab] OR Pakistan*[tiab] OR Paraguay*[tiab] OR Peru*[tiab] OR Philippin*[tiab] OR Pitcairn[tiab] OR Romania*[tiab] OR Rwanda*[tiab] OR Samoa*[tiab] OR Sao Tome[tiab] OR Principe[tiab] OR Senegal*[tiab] OR Serbia*[tiab] OR Sierra Leone*[tiab] OR Solomon Island*[tiab] OR Somalia* [tiab] OR South Africa*[tiab] OR Sri Lanka[tiab] OR St Helena[tiab] OR St Lucia[tiab] OR Saint Lucia[tiab] OR St Vincent[tiab] OR Saint Vincent[tiab] OR Grenad*[tiab] OR Sudan*[tiab] OR Suriname*[tiab] OR Swaziland*[tiab] OR Syria*[tiab] OR Tajik*[tiab] OR Tanzania*[tiab] OR Thai*[tiab] OR Timor*[tiab] OR Togo*[tiab] OR Tokelau[tiab] OR Tonga*[tiab] OR Tunisia*[tiab] OR Turkey[tiab] OR Turkish[tiab] OR Turkmen*[tiab] OR Tuvalu*[tiab] OR Uganda*[tiab] OR Ukrain*[tiab] OR Uzbeki*[tiab] OR Vanuatu*[tiab] OR Venezuela*[tiab] OR Vietnam*[tiab] OR Viet nam*[tiab] OR West Bank[tiab] OR Gaza*[tiab] OR Palestin*[tiab] OR Wallis and Futuna Island OR Yemen*[tiab] OR Zambia*[tiab] OR Zimbabw*[tiab] OR Western Sahara[tiab])
(5) Only human studies	NOT (Animals[Mesh] NOT (Animals[Mesh] AND Humans[Mesh]))
Search strategy	(1) And (2) And (3) And (4) and (5)

Supplemental Table 2. Characteristics of the eligible studies not included in the individual participant data meta-analysis[a]

Study	Country	Years of study	Sample size	Study design	Composition of supplements	Control	Timing of intervention initiation	Reason for lack of inclusion	Main findings from publications
Kæstel 2005[1]	Guinea-Bissau	2001-2002	2100	Parallel	Daily MMS tablet containing one RDA or two RDAs of 15 micronutrients	IFA containing 60 mg of iron and 0·4 mg of folic acid	From < 37 weeks gestation	Did not contribute individual-level data	MMS with two RDAs increased birthweight compared to IFA
Zagré 2007[2]	Niger	2004-2006	2550	Cluster	Daily MMS of the UNIMMAP formulation[b]	IFA containing 60 mg of iron and 0·4 mg of folic acid	From < 28 weeks gestation	Lack of gestational weight variable for the study to be included in the GWG Pooling Project	MMS increased mean birthweight compared to IFA
SUMMIT, 2008[3]	Indonesia	2001-2004	31290	Cluster	Daily MMS of the UNIMMAP formulation[b]	IFA containing 30 mg of iron and 0·4 mg of folic acid	From any gestational age	Did not contribute individual-level data	MMS reduced the risk of low birthweight compared to IFA
Sunawang, 2009[4]	Indonesia	2000-2003	843	Cluster	Daily MMS of the UNIMMAP formulation[b]	IFA containing 30 mg of iron and 0·25 mg of folic acid	From any gestational age	Not able to get in contact with the study team to invite data contribution	There were no significant differences between arms in low birthweight
Hanieh, 2013[5]	Vietnam	2010-2012	1258	Cluster	Twice-weekly MMS as two capsules/week; each capsule contained 60 mg of iron, 20 mg of zinc, 300 µg of iodine, 4 mg of copper, 130 µg of selenium, 1·6 mg of vitamin A, 2·8 mg of thiamine, 2·8 mg of riboflavin, 36 mg of niacin, 3·8 mg of vitamin B-6, 5·2 µg of vitamin B-12, 1·5 mg of folic acid, 140 mg of vitamin C, 400 IU of vitamin D, and 20 mg of vitamin E	Daily IFA tablet containing 60 mg of iron and 0·4 mg of folic acid; or twice-weekly IFA as two capsules/week, with each capsule containing 60 mg of iron and 1·5 mg of folic acid	From < 16 weeks gestation	Did not contribute individual-level data	Twice-weekly antenatal IFA or MMS did not produce a clinically important difference in birth weight when compared to daily IFA

[a] IFA, iron and folic acid; MMS, multiple micronutrient supplements; RDA, recommended dietary allowance; UNIMMAP, United Nations International Multiple Micronutrient Antenatal Preparation.

[b] The UNIMMAP formulation included 800 µg/d of vitamin A, 5 µg/d of vitamin D, 10 mg/d of vitamin E, 70 mg/d of vitamin C, 1·4 mg/d of vitamin B-1, 1·4 mg/d of vitamin B-2, 18 mg/d of niacin, 1·9 mg/d of vitamin B-6, 2·6 µg/d of vitamin B-12, 400 µg/d of folic acid, 30 mg/d of iron, 15 mg/d of zinc, 2 mg/d of copper, 65 µg/d of selenium, and 150 µg/d of iodine.

Supplemental Table 3. Missingness in outcome data in the included trials on multiple micronutrient supplements and small-quantity lipid-based nutrient supplements[1]

Study	Total sample size	Missing GA at birth[2]	Missing birthweight	Missing infant sex	Missing GA at birth, birthweight, or infant sex	Sample size in analysis
MMS analysis						
Christian, 2003	3008	270 (9·0)	390 (13·0)	99 (3·3)	625 (20·8)	2383
Ramakrishnan, 2003	874	221 (25·3)	234 (26·8)	218 (24·9)	238 (27·2)	636
Friis, 2004	1669	410 (24·6)	554 (33·2)	503 (30·1)	668 (40·0)	1001
Osrin, 2005	1200	80 (6·7)	172 (14·3)	77 (6·4)	196 (16·3)	1004
Fawzi, 2007	8536	811 (9·5)	670 (7·9)	138 (1·6)	1389 (16·3)	7147
Zeng, 2008	5877	1151 (19·6)	1493 (25·4)	1044 (17·8)	1613 (27·5)	4264
Roberfroid, 2008	1385	160 (11·6)	293 (21·2)	136 (9·8)	365 (26·4)	1020
Bhutta, 2009	2357	927 (39·3)	821 (34·8)	817 (34·7)	1038 (44·0)	1319
Persson, 2012	4472	1014 (22·7)	1206 (27·0)	840 (18·8)	1446 (32·3)	3026
Moore, 2012	869	74 (8·5)	183 (21·1)	50 (5·8)	203 (23·4)	666
West, 2014	25210	2118 (8·4)	6569 (26·1)	811 (3·2)	8095 (32·1)	17115
Ashorn, 2015	933	22 (2·4)	127 (13·6)	83 (8·9)	143 (15·3)	790
Adu-Afarwuah, 2015	880	52 (5·9)	140 (15·9)	101 (11·5)	153 (17·4)	727
Bliznashka, 2022	1669	147 (8·8)	2 (0·12)	0 (0)	149 (8·9)	1520
Total	58939	7457 (12·7)	12854 (21·8)	4917 (8·3)	16321 (27·7)	42618
SQ-LNS analysis						
Ashorn, 2015	936	27 (2·9)	118 (12·6)	76 (8·1)	138 (14·7)	798
Adu-Afarwuah, 2015	881	57 (6·5)	154 (17·5)	113 (12·8)	165 (18·7)	716
Matias, 2016	4011	608 (15·2)	562 (14·0)	266 (6·6)	895 (22·3)	3116
Hambidge, 2019	1809	113 (6·3)	95 (5·3)	1 (0·06)	193 (10·7)	1616
Total	7637	805 (10·5)	929 (12·2)	456 (6·0)	1391 (18·2)	6246

[1] The values are counts and percentages by study and by analysis. GA, gestational age; MMS, multiple micronutrient supplements; SQ-LNS, small-quantity lipid-based nutrient supplements.

[2] Including gestational age at birth less than 168 days or greater than 300 days, for which birthweight for sex and gestational age based on the INTERGROWTH-21st newborn size standards could not be derived.

Supplemental Table 4. Effects of prenatal multiple micronutrient supplements and small-quantity lipid-based nutrient supplements on newborn types based on the four-group categorization by potential effect modifiers[1]

	Newborn types based on the four-group categorization								
	Term-SGA			Preterm-nonSGA			Preterm-SGA		
	Number of studies	Pooled RR (95% CI)	*P* for interaction	Number of studies	Pooled RR (95% CI)	*P* for interaction	Number of studies	Pooled RR (95% CI)	*P* for interaction
MMS									
Maternal age, years									
< 20	14	0·94 (0·92, 0·97)	0·26	12	0·89 (0·80, 0·98)	0·40	10	0·70 (0·57, 0·86)	0·87
20-29	14	0·95 (0·92, 0·97)		13	0·93 (0·88, 0·98)		12	0·74 (0·62, 0·88)	
≥ 30	14	0·99 (0·94, 1·05)		13	0·97 (0·89, 1·06)		9	0·77 (0·55, 1·07)	
Parity									
0	14	0·93 (0·90, 0·95)	0·024	13	0·87 (0·81, 0·94)	0·044	12	0·63 (0·53, 0·75)	0·042
≥ 1	12	0·97 (0·94, 0·99)		12	0·96 (0·91, 1·01)		11	0·81 (0·69, 0·96)	
Gestational age at enrollment, weeks									
< 20	13	0·96 (0·94, 0·98)	0·089	13	0·92 (0·87, 0·97)	0·21	12	0·71 (0·63, 0·81)	0·25
≥ 20	11	0·90 (0·83, 0·96)		9	0·99 (0·89, 1·10)		6	0·92 (0·61, 1·40)	
Early-pregnancy BMI, kg/m^2									
< 18·5	13	0·97 (0·94, 1·00)	0·48	12	0·85 (0·77, 0·94)	0·33	7	0·77 (0·63, 0·96)	0·58
18·5 to < 25·0	14	0·94 (0·92, 0·97)		14	0·93 (0·88, 0·98)		10	0·68 (0·57, 0·80)	
≥ 25·0	14	0·97 (0·85, 1·12)		8	0·95 (0·82, 1·10)		4	0·83 (0·33, 2·09)	
Maternal anemia									
No anemia	12	1·02 (0·97, 1·07)	0·064	12	0·89 (0·78, 1·02)	0·74	8	0·76 (0·55, 1·06)	0·79
Mild anemia	12	0·93 (0·86, 1·01)		11	0·97 (0·80, 1·18)		6	0·90 (0·44, 1·82)	
Moderate to severe anemia	12	0·91 (0·80, 1·03)		11	0·95 (0·80, 1·12)		5	0·97 (0·50, 1·88)	
SQ-LNS									
Maternal age, years									
< 20	4	0·96 (0·88, 1·04)	0·39	4	1·02 (0·81, 1·27)	0·020	2	0·80 (0·50, 1·28)	0·61
20-29	4	0·93 (0·89, 0·97)		4	0·86 (0·72, 1·02)		4	1·17 (0·64, 2·13)	
≥ 30	4	0·85 (0·73, 0·99)		4	0·64 (0·50, 0·81)		2	1·09 (0·24, 4·85)	
Parity									
0	4	0·92 (0·87, 0·98)	0·33	4	1·18 (0·98, 1·43)	0·0010	3	0·99 (0·53, 1·85)	0·30
≥ 1	3	0·89 (0·86, 0·92)		3	0·81 (0·72, 0·91)		3	0·64 (0·37, 1·10)	
Gestational age at enrollment, weeks									
< 20	4	0·89 (0·88, 0·91)	0·023	4	0·86 (0·77, 0·96)	0·83	4	0·73 (0·54, 0·99)	0·012
≥ 20	3	1·12 (0·92, 1·36)		3	0·93 (0·45, 1·92)		1	4·75 (1·15, 19·59)	
Early-pregnancy BMI, kg/m^2									
< 18·5	4	0·84 (0·82, 0·87)	< 0·0001	4	0·91 (0·73, 1·13)	0·59	2	0·75 (0·48, 1·17)	0·43
18·5 to < 25·0	4	0·93 (0·92, 0·93)		4	0·81 (0·67, 0·99)		3	0·95 (0·64, 1·42)	
≥ 25·0	4	0·83 (0·72, 0·97)		4	1·03 (0·60, 1·76)		0	NA	
Maternal anemia									
No anemia	4	1·01 (0·95, 1·07)	< 0·0001	4	0·95 (0·85, 1·05)	< 0·0001	4	1·11 (0·56, 2·19)	< 0·0001
Mild anemia	4	0·75 (0·67, 0·85)		4	0·76 (0·62, 0·91)		3	0·22 (0·16, 0·32)	
Moderate to severe anemia	4	0·83 (0·71, 0·98)		4	0·49 (0·37, 0·64)		1	1·18 (0·64, 2·19)	

[1] Values are pooled risk ratios and 95% confidence intervals from fixed-effect meta-analytical models comparing MMS or SQ-LNS with control. The study-specific estimates (omitted from the table for brevity) were calculated using log-binomial or modified Poisson models. Term-nonSGA was used as the reference group in all models. CI, confidence interval; MMS, multiple micronutrient supplements; nonSGA, not small for gestational age; RR, risk ratio; SGA, small for gestational age; SQ-LNS, small-quantity lipid-based nutrient supplements.

Supplemental Table 5. Effects of prenatal multiple micronutrient supplements and small-quantity lipid-based nutrient supplements on newborn types based on the ten-group categorization, after adjusting for covariates when estimating the study-specific estimates[1]

	**Newborn types based on the ten-group categorization**									
	**Term-AGA-nonLBW**	**Preterm-SGA-LBW**	**Preterm-AGA-LBW**	**Preterm-LGA-LBW**	**Term-SGA-LBW**	**Term-AGA-LBW**	**Preterm-AGA-nonLBW**	**Preterm-LGA-nonLBW**	**Term-SGA-nonLBW**	**Term-LGA-nonLBW**
	**RR (95% CI)**	**RR (95% CI)**	**RR (95% CI)**	**RR (95% CI)**	**RR (95% CI)**	**RR (95% CI)**	**RR (95% CI)**	**RR (95% CI)**	**RR (95% CI)**	**RR (95% CI)**
MMS										
Christian, 2003[2]	Reference	1·26 (0·51, 3·12)	0·87 (0·37, 2·09)	5·21 (0·74, 36·61)	1·31 (1·09, 1·57)	1·36 (0·33, 5·65)	1·08 (0·52, 2·24)	1·89 (0·78, 4·61)	1·22 (0·89, 1·68)	5·51 (0·29, 104·74)
Ramakrishnan, 2003	Reference	5·75 (0·11, 303·44)	1·79 (0·33, 9·74)	Zero events	0·55 (0·23, 1·28)	Zero events	2·03 (0·15, 26·64)	Zero events	0·78 (0·55, 1·10)	0·61 (0·09, 3·98)
Friis, 2004	Reference	4·08 (0·48, 34·75)	1·12 (0·35, 3·58)	Low events (*n* = 1)	0·64 (0·30, 1·37)	Zero events	1·17 (0·53, 2·59)	1·21 (0·57, 2·58)	1·24 (0·75, 2·06)	2·87 (0·55, 14·98)
Osrin, 2005	Reference	1·09 (0·52, 2·27)	0·54 (0·27, 1·10)	Zero events	0·70 (0·53, 0·91)	1·48 (0·13, 16·54)	0·84 (0·32, 2·19)	0·85 (0·05, 13·86)	0·90 (0·74, 1·09)	1·66 (0·42, 6·61)
Fawzi, 2007	Reference	1·03 (0·57, 1·89)	0·74 (0·54, 1·01)	0·80 (0·43, 1·49)	0·65 (0·50, 0·85)	Low events (*n* = 1)	1·06 (0·88, 1·28)	1·02 (0·87, 1·20)	0·82 (0·73, 0·93)	0·95 (0·78, 1·16)
Zeng, 2008[2]	Reference	0·33 (0·08, 1·44)	0·92 (0·43, 2·00)	Low events (*n* = 5)	1·10 (0·73, 1·66)	Low events (*n* = 3)	0·78 (0·48, 1·26)	1·35 (0·81, 2·26)	1·00 (0·84, 1·18)	1·20 (0·92, 1·55)
Roberfroid, 2008	Reference	1·87 (0·45, 7·78)	1·03 (0·49, 2·16)	0·15 (0·02, 0·87)	0·97 (0·65, 1·44)	Low events (*n* = 2)	0·76 (0·39, 1·50)	1·84 (0·84, 4·02)	0·95 (0·72, 1·27)	1·85 (0·51, 6·72)
Bhutta, 2009[2]	Reference	0·61 (0·24, 1·51)	0·94 (0·59, 1·52)	0·66 (0·34, 1·29)	0·95 (0·72, 1·26)	Low events (*n* = 2)	0·88 (0·58, 1·34)	0·97 (0·83, 1·12)	0·97 (0·73, 1·29)	1·31 (0·89, 1·93)
Persson, 2012	Reference	0·82 (0·47, 1·41)	0·82 (0·47, 1·43)	Zero events	0·99 (0·89, 1·11)	0·33 (0·04, 2·75)	1·23 (0·48, 3·18)	Zero events	1·03 (0·94, 1·13)	0·67 (0·07, 6·43)
Moore, 2012	Reference	Low events (*n* = 1)	1·43 (0·19, 11·05)	Zero events	0·78 (0·48, 1·26)	Low events (*n* = 1)	Low events (*n* = 1)	0·51 (0·05, 5·68)	0·97 (0·74, 1·26)	1·71 (0·58, 5·03)
West, 2014[2]	Reference	0·69 (0·60, 0·79)	0·80 (0·71, 0·90)	0·48 (0·24, 0·95)	0·91 (0·87, 0·94)	1·15 (0·79, 1·69)	0·82 (0·72, 0·94)	1·02 (0·82, 1·27)	0·98 (0·93, 1·02)	1·53 (0·79, 2·96)
Ashorn, 2015	Reference	0·62 (0·14, 2·68)	0·93 (0·40, 2·18)	Low events (*n* = 1)	1·05 (0·68, 1·61)	Low events (*n* = 1)	0·76 (0·28, 2·06)	1·10 (0·26, 4·62)	0·71 (0·52, 0·96)	0·87 (0·41, 1·81)
Adu-Afarwuah, 2015	Reference	0·72 (0·16, 3·23)	0·30 (0·09, 1·04)	Low events (*n* = 1)	0·73 (0·43, 1·24)	Low events (*n* = 1)	0·74 (0·33, 1·67)	Low events (*n* = 6)	1·02 (0·59, 1·78)	1·47 (0·84, 2·58)
Bliznashka, 2022[2]	Reference	1·74 (0·54, 5·60)	0·97 (0·55, 1·74)	0·62 (0·17, 2·23)	0·85 (0·49, 1·47)	Low events (*n* = 1)	0·85 (0·67, 1·09)	1·18 (0·99, 1·42)	1·08 (0·73, 1·61)	1·51 (0·68, 3·31)
Pooled, fixed-effect	Reference	0·74 (0·65, 0·84)	0·80 (0·73, 0·89)	0·65 (0·46, 0·92)	0·92 (0·89, 0·95)	1·13 (0·79, 1·62)	0·89 (0·81, 0·97)	1·06 (0·97, 1·15)	0·97 (0·93, 1·00)	1·13 (0·99, 1·29)
Pooled, random-effects	Reference	0·81 (0·65, 1·00)	0·80 (0·72, 0·90)	0·65 (0·31, 1·38)	0·91 (0·80, 1·03)	1·13 (0·63, 2·02)	0·89 (0·80, 0·98)	1·06 (0·96, 1·16)	0·95 (0·89, 1·02)	1·13 (0·98, 1·31)
Heterogeneity										
I^2		9·05%	0·00%	39·53%	59·23%	0·00%	0·00%	0·00%	29·94%	0·00%
P		0·36	0·90	0·14	0·0025	0·70	0·86	0·63	0·14	0·64
SQ-LNS										
Ashorn, 2015	Reference	Low events (*n* = 4)	0·92 (0·39, 2·17)	Zero events	0·92 (0·59, 1·43)	Low events (*n* = 1)	0·48 (0·17, 1·36)	0·48 (0·09, 2·59)	0·80 (0·60, 1·07)	0·97 (0·48, 1·95)
Adu-Afarwuah, 2015	Reference	0·73 (0·17, 3·12)	0·33 (0·09, 1·19)	Low events (*n* = 1)	0·33 (0·16, 0·69)	Zero events	1·38 (0·66, 2·91)	1·15 (0·39, 3·37)	1·04 (0·60, 1·81)	1·54 (0·89, 2·64)
Matias, 2016[2]	Reference	0·66 (0·29, 1·50)	0·96 (0·66, 1·41)	0·60 (0·29, 1·25)	0·90 (0·79, 1·03)	0·47 (0·12, 1·83)	0·99 (0·73, 1·33)	1·04 (0·69, 1·58)	0·93 (0·82, 1·05)	1·69 (0·36, 8·01)
Hambidge, 2019[3]	Reference	1·30 (0·24, 7·09)	0·66 (0·21, 2·06)	Zero events	0·72 (0·67, 0·77)	1·45 (0·89, 2·37)	0·82 (0·26, 2·64)	0·63 (0·39, 1·03)	1·05 (0·87, 1·27)	Low events (*n* = 3)
Pooled, fixed-effect	Reference	0·74 (0·38, 1·44)	0·87 (0·63, 1·20)	Cannot synthesize	0·75 (0·71, 0·80)	1·27 (0·80, 2·02)	0·97 (0·75, 1·27)	0·85 (0·63, 1·14)	0·95 (0·86, 1·04)	1·32 (0·87, 1·99)
Pooled, random-effects	Reference	0·74 (0·17, 3·19)	0·87 (0·51, 1·47)	Cannot synthesize	0·77 (0·48, 1·24)	0·99 (0, 847.36)	0·97 (0·64, 1·49)	0·85 (0·51, 1·40)	0·95 (0·81, 1·11)	1·32 (0·53, 3·26)
Heterogeneity										
I^2		0·00%	0·00%	Cannot synthesize	78·83%	56·88%	0·00%	3·43%	0·00%	0·00%
P		0·78	0·43	Cannot synthesize	0·0027	0·13	0·44	0·38	0·42	0·56

[1] Value are risk ratios and 95% confidence intervals comparing MMS or SQ-LNS with control, computed using log-binomial or modified Poisson models. The covariates included maternal age, parity, gestational age at study enrollment, early-pregnancy body mass index, and maternal anemia at baseline (availability of these covariates varied across studies, and the covariates available in the study were adjusted). Estimates are not available for models marked with zero events. Estimates are also not available for models marked with low events (with the number of outcome events shown in paratheses) due to failure of model convergence. AGA, appropriate for gestational age; CI, confidence interval; LBW, low birthweight; LGA, large for gestational age; MMS, multiple micronutrient supplements; nonLBW, not low birthweight; RR, risk ratio; SGA, small for gestational age; SQ-LNS, small-quantity lipid-based nutrient supplements.

[2] For cluster-randomized trials, Poisson models with cluster-robust standard errors were used.

[3] The study arm that started supplementation from the preconceptional period was excluded as the analysis focused on the effect of prenatal supplementation initiated during pregnancy. Poisson models with cluster-robust standard errors were used to account for country.

Supplemental Table 6. Effects of prenatal multiple micronutrient supplements and small-quantity lipid-based nutrient supplements on newborn types based on the four-group categorization, after adjusting for covariates when estimating the study-specific estimates[1]

	Newborn types based on the four-group categorization			
	**Term-nonSGA**	**Term-SGA**	**Preterm-nonSGA**	**Preterm-SGA**
	**RR (95% CI)**	**RR (95% CI)**	**RR (95% CI)**	**RR (95% CI)**
MMS				
Christian, 2003[2]	Reference	1·17 (1·01, 1·35)	1·17 (0·76, 1·82)	1·32 (0·53, 3·30)
Ramakrishnan, 2003	Reference	0·76 (0·56, 1·03)	1·80 (0·45, 7·12)	5·30 (0·10, 270·29)
Friis, 2004	Reference	1·02 (0·69, 1·50)	1·06 (0·66, 1·69)	Low events (*n* = 2)
Osrin, 2005	Reference	0·85 (0·74, 0·98)	0·64 (0·37, 1·09)	1·09 (0·52, 2·27)
Fawzi, 2007	Reference	0·80 (0·72, 0·89)	0·99 (0·89, 1·10)	1·03 (0·57, 1·89)
Zeng, 2008[2]	Reference	1·00 (0·86, 1·17)	0·93 (0·67, 1·29)	0·33 (0·08, 1·43)
Roberfroid, 2008	Reference	0·96 (0·78, 1·19)	1·00 (0·69, 1·44)	1·85 (0·45, 7·71)
Bhutta, 2009[2]	Reference	0·95 (0·79, 1·14)	0·93 (0·80, 1·07)	0·59 (0·23, 1·46)
Persson, 2012	Reference	1·01 (0·96, 1·08)	0·95 (0·59, 1·52)	0·83 (0·48, 1·43)
Moore, 2012	Reference	0·93 (0·75, 1·15)	0·71 (0·20, 2·57)	Low events (*n* = 1)
West, 2014[2]	Reference	0·95 (0·93, 0·98)	0·85 (0·79, 0·92)	0·68 (0·59, 0·79)
Ashorn, 2015	Reference	0·83 (0·66, 1·04)	0·92 (0·53, 1·58)	0·62 (0·14, 2·70)
Adu-Afarwuah, 2015	Reference	0·85 (0·59, 1·22)	0·43 (0·23, 0·79)	0·70 (0·16, 3·09)
Bliznashka, 2022[2]	Reference	0·99 (0·67, 1·44)	1·02 (0·90, 1·15)	Low events (*n* = 8)
Pooled, fixed-effect	Reference	0·95 (0·93, 0·97)	0·93 (0·88, 0·97)	0·72 (0·64, 0·82)
Pooled, random-effects	Reference	0·94 (0·88, 1·00)	0·93 (0·86, 1·02)	0·72 (0·62, 0·84)
Heterogeneity				
I^2		55·73%	29·77%	0·00%
P		0·0058	0·14	0·53
SQ-LNS				
Ashorn, 2015	Reference	0·85 (0·68, 1·06)	0·68 (0·38, 1·22)	Low events (*n* = 4)
Adu-Afarwuah, 2015	Reference	0·66 (0·44, 0·99)	0·90 (0·54, 1·50)	0·70 (0·16, 3·09)
Matias, 2016[2]	Reference	0·94 (0·87, 1·01)	0·97 (0·80, 1·18)	0·65 (0·28, 1·49)
Hambidge, 2019[3]	Reference	0·90 (0·86, 0·95)	0·70 (0·29, 1·70)	1·30 (0·23, 7·15)
Pooled, fixed-effect	Reference	0·91 (0·87, 0·94)	0·92 (0·78, 1·10)	0·73 (0·38, 1·43)
Pooled, random-effects	Reference	0·91 (0·84, 0.98)	0·92 (0·70, 1·22)	0·73 (0·17, 3·17)
Heterogeneity				
I^2		10·35%	0·00%	0·00%
P		0·34	0·63	0·77

[1] Value are risk ratios and 95% confidence intervals comparing MMS or SQ-LNS with control, computed using log-binomial or modified Poisson models. The covariates included maternal age, parity, gestational age at study enrollment, early-pregnancy body mass index, and maternal anemia at baseline (availability of these covariates varied across studies, and the covariates available in the study were adjusted). Estimates are not available for models marked with low events (with the number of outcome events shown in paratheses) due to failure of model convergence. CI, confidence interval; LGA, large for gestational age; MMS, multiple micronutrient supplements; nonSGA, not small for gestational age; RR, risk ratio; SGA, small for gestational age; SQ-LNS, small-quantity lipid-based nutrient supplements.
[2] For cluster-randomized trials, Poisson models with cluster-robust standard errors were used.
[3] The study arm that started supplementation from the preconceptional period was excluded as the analysis focused on the effect of prenatal supplementation initiated during pregnancy. Poisson models with cluster-robust standard errors were used to account for country.

Supplemental Table 7. Effects of prenatal multiple micronutrient supplements and small-quantity lipid-based nutrient supplements on newborn types based on the ten-group categorization when restricting to studies with ultrasound-based measures of gestational age[1]

	**Newborn types based on the ten-group categorization**									
	**Term-AGA-nonLBW**	**Preterm-SGA-LBW**	**Preterm-AGA-LBW**	**Preterm-LGA-LBW**	**Term-SGA-LBW**	**Term-AGA-LBW**	**Preterm-AGA-nonLBW**	**Preterm-LGA-nonLBW**	**Term-SGA-nonLBW**	**Term-LGA-nonLBW**
	**RR (95% CI)**	**RR (95% CI)**	**RR (95% CI)**	**RR (95% CI)**	**RR (95% CI)**	**RR (95% CI)**	**RR (95% CI)**	**RR (95% CI)**	**RR (95% CI)**	**RR (95% CI)**
MMS (*N* = 5526)										
Osrin, 2005	Reference	1·00 (0·47, 2·12)	0·60 (0·30, 1·20)	Zero events	0·68 (0·52, 0·90)	1·70 (0·16, 18·68)	0·86 (0·33, 2·25)	0·86 (0·05, 13·60)	0·89 (0·73, 1·08)	1·69 (0·43, 6·70)
Roberfroid, 2008	Reference	1·00 (0·38, 2·63)	1·08 (0·63, 1·86)	0·67 (0·19, 2·35)	0·98 (0·69, 1·40)	Low events (*n* = 3)	1·00 (0·56, 1·79)	1·70 (0·85, 3·39)	0·94 (0·72, 1·22)	2·30 (0·60, 8·82)
Bhutta, 2009[2]	Reference	0·61 (0·22, 1·69)	1·02 (0·64, 1·61)	0·76 (0·40, 1·44)	1·00 (0·75, 1·34)	Low events (*n* = 2)	0·85 (0·57, 1·27)	1·00 (0·82, 1·22)	0·96 (0·74, 1·25)	1·44 (0·88, 2·37)
Moore, 2012	Reference	Low events (*n* = 1)	0·93 (0·19, 4·56)	Zero events	0·83 (0·51, 1·35)	Low events (*n* = 1)	Low events (*n* = 1)	0·47 (0·04, 5·12)	0·98 (0·76, 1·28)	1·82 (0·63, 5·25)
Ashorn, 2015	Reference	0·47 (0·12, 1·87)	0·86 (0·39, 1·92)	Low events (*n* = 1)	1·03 (0·68, 1·57)	Low events (*n* = 1)	0·66 (0·26, 1·71)	0·93 (0·24, 3·69)	0·74 (0·55, 0·99)	0·81 (0·38, 1·71)
Adu-Afarwuah, 2015	Reference	1·38 (0·40, 4·85)	0·52 (0·19, 1·38)	0·93 (0·06, 14·78)	0·65 (0·39, 1·08)	Low events (*n* = 1)	0·79 (0·36, 1·74)	Low events (*n* = 6)	0·93 (0·54, 1·61)	1·45 (0·82, 2·55)
Pooled, fixed-effect	Reference	0·87 (0·56, 1·37)	0·88 (0·67, 1·16)	0·75 (0·43, 1·31)	0·85 (0·74, 0·99)	Cannot synthesize	0·86 (0·65, 1·13)	1·03 (0·86, 1·25)	0·90 (0·81, 1·01)	1·38 (1·02, 1·87)
Pooled, random-effects	Reference	0·87 (0·46, 1·65)	0·88 (0·61, 1·26)	0·75 (0·22, 2·56)	0·85 (0·69, 1·06)	Cannot synthesize	0·86 (0·58, 1·27)	1·03 (0·79, 1·35)	0·90 (0·78, 1·04)	1·38 (0·93, 2·06)
SQ-LNS (*N* = 1924)										
Ashorn, 2015	Reference	0·79 (0·24, 2·56)	1·02 (0·48, 2·20)	Zero events	1·04 (0·69, 1·58)	Low events (*n* = 1)	0·48 (0·17, 1·39)	0·48 (0·09, 2·58)	0·83 (0·63, 1·11)	1·01 (0·50, 2·05)
Adu-Afarwuah, 2015	Reference	0·96 (0·24, 3·80)	0·27 (0·08, 0·96)	Low events (*n* = 1)	0·35 (0·18, 0·67)	Zero events	1·24 (0·62, 2·50)	1·12 (0·38, 3·28)	0·96 (0·56, 1·66)	1·63 (0·94, 2·84)
Hambidge, 2019[3]	Reference	Low events (*n* = 2)	1·46 (0·49, 4·34)	Zero events	0·81 (0·45, 1·49)	0·93 (0·13, 6·51)	1·54 (0·63, 3·80)	0·74 (0·28, 1·92)	0·92 (0·61, 1·40)	Low events (*n* = 3)
Pooled, fixed-effect	Reference	0·86 (0·35, 2·10)	0·86 (0·49, 1·52)	Cannot synthesize	0·77 (0·57, 1·05)	Cannot synthesize	1·08 (0·66, 1·77)	0·81 (0·42, 1·56)	0·88 (0·71, 1·09)	1·36 (0·88, 2·10)
Pooled, random-effects	Reference	0·86 (0·25, 2·92)	0·80 (0·10, 6·20)	Cannot synthesize	0·69 (0·17, 2·83)	Cannot synthesize	1·05 (0·26, 4·24)	0·81 (0·19, 3·42)	0·88 (0·55, 1·41)	1·35 (0·07, 26·19)

[1] Values are risk ratios and 95% confidence intervals comparing MMS or SQ-LNS with control, computed using log-binomial or modified Poisson models. Estimates are not available for models marked with zero events. Estimates are also not available for models marked with low events (with the number of outcome events shown in paratheses) due to failure of model convergence. AGA, appropriate for gestational age; CI, confidence interval; LBW, low birthweight; LGA, large for gestational age; MMS, multiple micronutrient supplements; nonLBW, not low birthweight; RR, risk ratio; SGA, small for gestational age; SQ-LNS, small-quantity lipid-based nutrient supplements.

[2] For cluster-randomized trials, Poisson models with cluster-robust standard errors were used.

[3] The study arm that started supplementation from the preconceptional period was excluded as the analysis focused on the effect of prenatal supplementation initiated during pregnancy. Data from the Democratic Republic of the Congo were excluded due to a lack of ultrasound-based measures of gestational age. Data from the India and Pakistan sites were also removed because a considerable proportion of the participants in these two sites received additional lipid-based nutrient supplements (302 kcal and did not qualify as SQ-LNS) because of low body mass index or inadequate gestational weight gain. Therefore, only data from the Guatemala site were retained in the analysis. Poisson models with cluster-robust standard errors were used to account for country.

Supplemental Table 8. Effects of prenatal multiple micronutrient supplements and small-quantity lipid-based nutrient supplements on newborn types based on the four-group categorization when restricting to studies with ultrasound-based measures of gestational age[1]

	Newborn types based on the four-group categorization			
	**Term-nonSGA**	**Term-SGA**	**Preterm-nonSGA**	**Preterm-SGA**
	**RR (95% CI)**	**RR (95% CI)**	**RR (95% CI)**	**RR (95% CI)**
MMS (*N* = 5526)				
Osrin, 2005	Reference	0·84 (0·73, 0·97)	0·69 (0·40, 1·17)	0·99 (0·46, 2·09)
Roberfroid, 2008	Reference	0·96 (0·79, 1·17)	1·11 (0·82, 1·52)	1·00 (0·38, 2·62)
Bhutta, 2009[2]	Reference	0·96 (0·79, 1·16)	0·94 (0·82, 1·08)	0·58 (0·21, 1·64)
Moore, 2012	Reference	0·94 (0·76, 1·17)	0·62 (0·18, 2·16)	Low events (*n* = 1)
Ashorn, 2015	Reference	0·85 (0·68, 1·07)	0·84 (0·49, 1·44)	0·48 (0·12, 1·88)
Adu-Afarwuah, 2015	Reference	0·76 (0·53, 1·09)	0·55 (0·31, 0·96)	1·34 (0·38, 4·69)
Pooled, fixed-effect	Reference	0·89 (0·82, 0·97)	0·92 (0·82, 1·03)	0·86 (0·55, 1·35)
Pooled, random-effects	Reference	0·89 (0·80, 0·99)	0·89 (0·70, 1·13)	0·86 (0·46, 1·63)
SQ-LNS (*N* = 1924)				
Ashorn, 2015	Reference	0·91 (0·73, 1·13)	0·74 (0·42, 1·30)	0·79 (0·24, 2·55)
Adu-Afarwuah, 2015	Reference	0·61 (0·41, 0·90)	0·82 (0·50, 1·33)	0·92 (0·23, 3·64)
Hambidge, 2019[3]	Reference	0·89 (0·64, 1·22)	1·14 (0·68, 1·93)	Low events (*n* = 2)
Pooled, fixed-effect	Reference	0·84 (0·71, 0·99)	0·89 (0·66, 1·20)	0·84 (0·34, 2·05)
Pooled, random-effects	Reference	0·82 (0·50, 1·35)	0·89 (0·46, 1·72)	0·84 (0·32, 2·19)

[1] Values are risk ratios and 95% confidence intervals comparing MMS or SQ-LNS with control, computed using log-binomial or modified Poisson models. Estimates are not available for models marked with low events (with the number of outcome events shown in paratheses) due to failure of model convergence. CI, confidence interval; MMS, multiple micronutrient supplements; nonSGA, not small for gestational age; RR, risk ratio; SGA, small for gestational age; SQ-LNS, small-quantity lipid-based nutrient supplements.
[2] For cluster-randomized trials, Poisson models with cluster-robust standard errors were used.
[3] The study arm that started supplementation from the preconceptional period was excluded as the analysis focused on the effect of prenatal supplementation initiated during pregnancy. Data from the Democratic Republic of the Congo were excluded due to a lack of ultrasound-based measures of gestational age. Data from the India and Pakistan sites were also removed because a considerable proportion of the participants in these two sites received additional lipid-based nutrient supplements (302 kcal and did not qualify as SQ-LNS) because of low body mass index or inadequate gestational weight gain. Therefore, only data from the Guatemala site were retained in the analysis. Poisson models with cluster-robust standard errors were used to account for country.

Supplemental Table 9. Effects of prenatal multiple micronutrient supplements and small-quantity lipid-based nutrient supplements on neonatal mortality[1]

	**Neonatal mortality**	
	**RR (95% CI)**	
MMS Studies	Control	MMS
Christian, 2003[2]	Reference	1·74 (1·06, 2·86)
Ramakrishnan, 2003	Reference	0·54 (0·10, 2·94)
Friis, 2004	Reference	1·15 (0·42, 3·14)
Osrin, 2005	Reference	1·53 (0·72, 3·23)
Fawzi, 2007	Reference	0·87 (0·67, 1·13)
Zeng, 2008[2]	Reference	0·81 (0·47, 1·38)
Roberfroid, 2008	No neonatal mortality data	No neonatal mortality data
Bhutta, 2009[2]	Reference	1·41 (0·94, 2·11)
Persson, 2012	Reference	1·10 (0·56, 2·14)
Moore, 2012	Reference	1·88 (0·57, 6·20)
West, 2014[2]	Reference	1·12 (0·96, 1·29)
Ashorn, 2015	Reference	0·82 (0·42, 1·60)
Adu-Afarwuah, 2015	Reference	1·00 (0·25, 3·99)
Bliznashka, 2022[2]	Reference	Low events (*n* = 1)
Pooled, fixed-effect	Reference	1·10 (0·98, 1·22)
Pooled, random-effects	Reference	1·10 (0·95, 1·28)
I^2		9%
SQ-LNS Studies	Control	SQ-LNS
Ashorn, 2015	Reference	0·62 (0·30, 1·29)
Adu-Afarwuah, 2015	Reference	2·00 (0·61, 6·61)
Matias, 2016[2]	Reference	0·89 (0·50, 1·59)
Hambidge, 2019[3]	Reference	1·57 (0·82, 3·01)
Pooled, fixed-effect	Reference	1·04 (0·73, 1·48)
Pooled, random-effects	Reference	1·06 (0·49, 2·31)
I^2		39%

[1] Values are risk ratios and 95% confidence intervals of neonatal mortality (defined as death within the first 28 days from birth), comparing MMS or SQ-LNS with control. The values are computed using log-binomial or modified Poisson models. CI, confidence interval; MMS, multiple micronutrient supplements; RR, risk ratio; SQ-LNS, small-quantity lipid-based nutrient supplements.
[2] For cluster-randomized trials, Poisson models with cluster-robust standard errors were used.
[3] The study arm that started supplementation from the preconceptional period was excluded as the analysis focused on the effect of prenatal supplementation initiated during pregnancy. Poisson models with cluster-robust standard errors were used to account for country.

Supplemental Table 10. Risk of bias of the included studies[1]

	Bias arising from the randomization process	Bias due to deviations from intended intervention	Bias due to missing outcome data	Bias in measurement of the outcome	Bias in selection of the reported result	For cluster RCT: bias arising from the timing of identification and recruitment of individual participants in relation to timing of randomization	Overall risk of bias
Christian, 2003	Low	Low	Some concerns	Some concerns	Low	Low	Some concerns
Ramakrishnan, 2003	Low	Low	Some concerns	Some concerns	Low	NA	Some concerns
Friis, 2004	Low	Low	Some concerns	Some concerns	Low	NA	Some concerns
Osrin, 2005	Low	Low	Low	Low	Low	NA	Low
Fawzi, 2007	Low	Low	Low	Low	Low	NA	Low
Zeng, 2008	Low	Low	Some concerns	Some concerns	Low	Low	Some concerns
Roberfroid, 2008	Low	Low	Some concerns	Low	Low	NA	Some concerns
Bhutta, 2009	Low	Low	Some concerns	Some concerns	Low	Low	Some concerns
Persson, 2012	Low	Low	Some concerns	Some concerns	Low	NA	Some concerns
Moore, 2012	Low	Low	Some concerns	Low	Low	NA	Some concerns
West, 2014	Low	Low	Some concerns	Some concerns	Low	Low	Some concerns
Ashorn, 2015	Low	Low	Low	Low	Low	NA	Low
Adu-Afarwuah, 2015	Low	Low	Low	Low	Low	NA	Low
Matias, 2016	Low	Low	Some concerns	Some concerns	Low	Low	Some concerns
Hambidge, 2019	Low	Low	Low	Low	Low	NA	Low
Bliznashka, 2022	Low	Low	Low	Some concerns	Low	Low	Some concerns

RCT, randomized controlled trial.

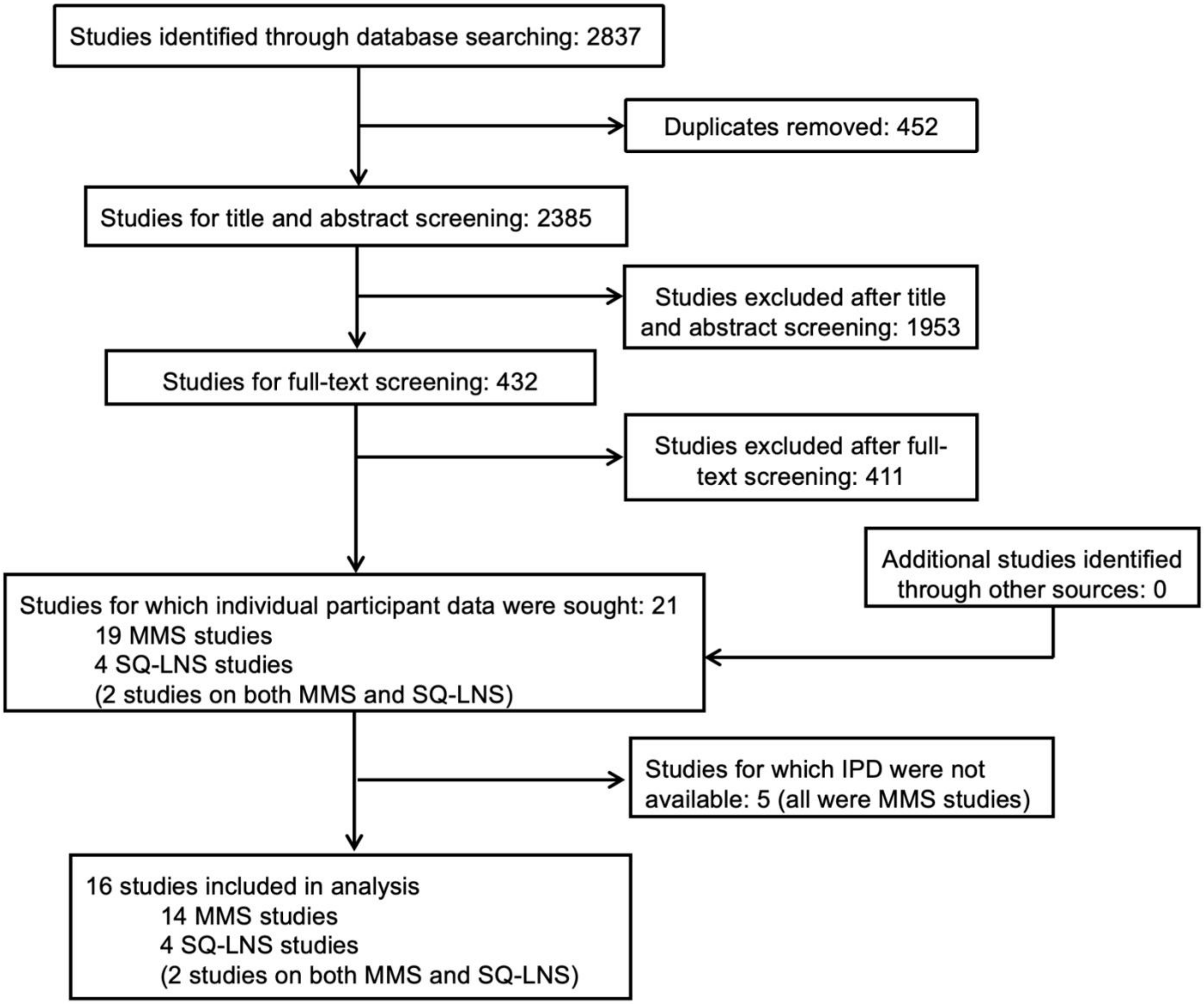

Supplemental Figure 1. PRISMA flow diagram for the individual participant data meta-analysis on the effects of prenatal nutritional supplements on small vulnerable newborn types in low- and middle-income countries. MMS, multiple micronutrient supplements; SQ-LNS, small-quantity lipid-based nutrient supplements.

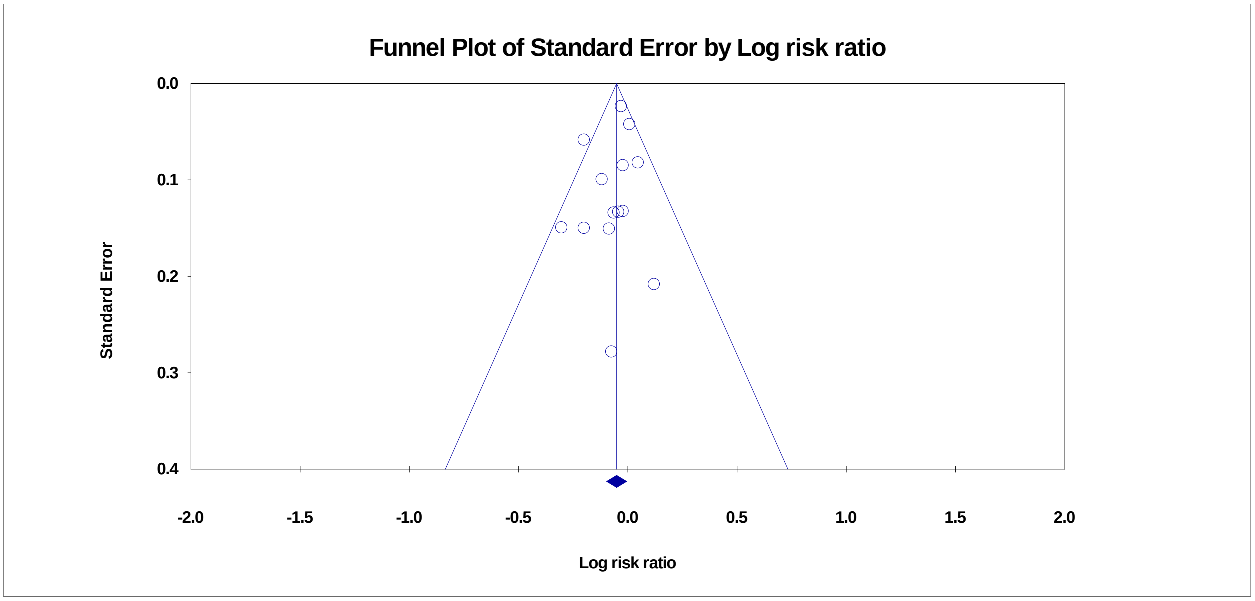

Supplemental Figure 2. Funnel plot for the effect of prenatal multiple micronutrient supplements on the small vulnerable newborn type of term-SGA-nonLBW.

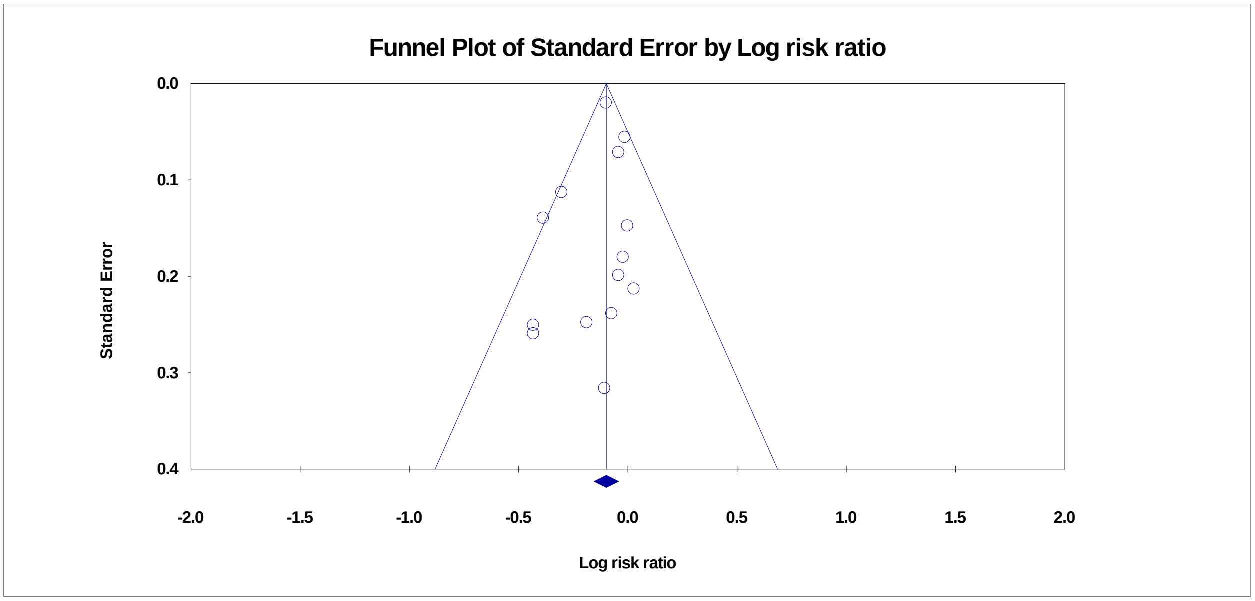

Supplemental Figure 3. Funnel plot for the effect of prenatal multiple micronutrient supplements on the small vulnerable newborn type of term-SGA-LBW.

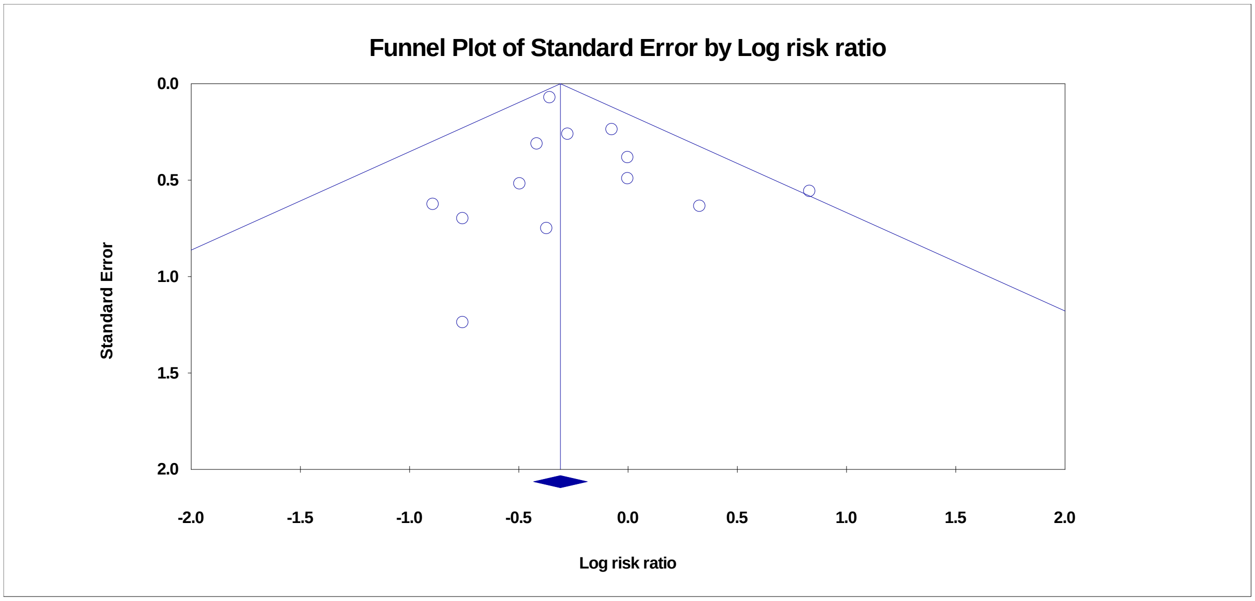

Supplemental Figure 4. Funnel plot for the effect of prenatal multiple micronutrient supplements on the small vulnerable newborn type of preterm-SGA-LBW.

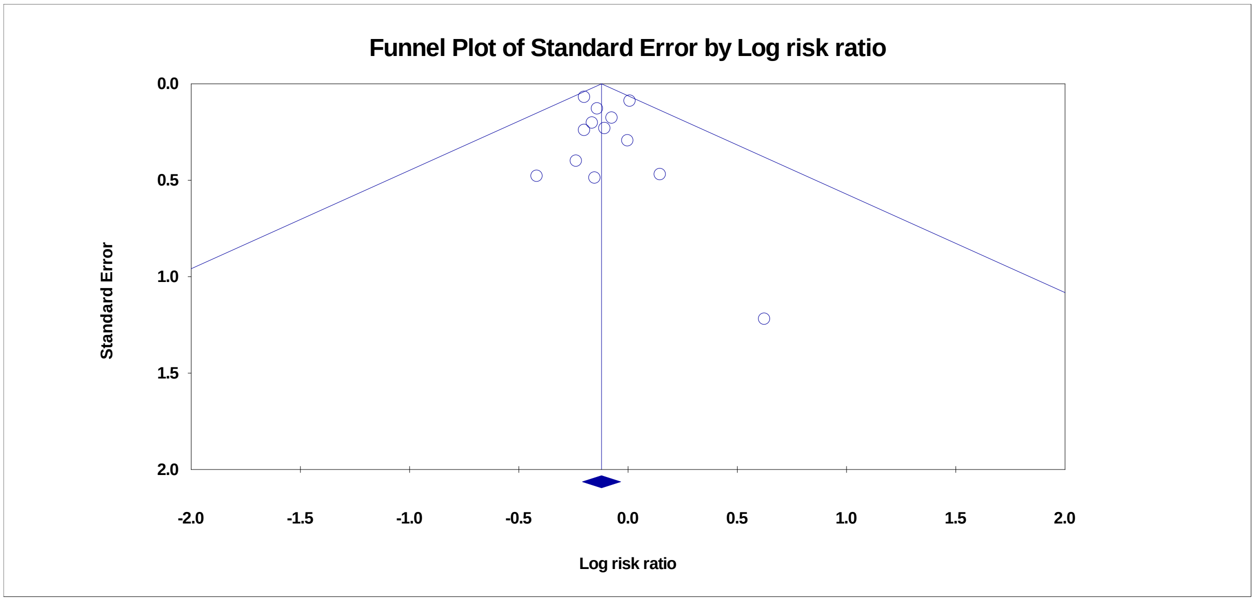

Supplemental Figure 5. Funnel plot for the effect of prenatal multiple micronutrient supplements on the small vulnerable newborn type of preterm-AGA-nonLBW.

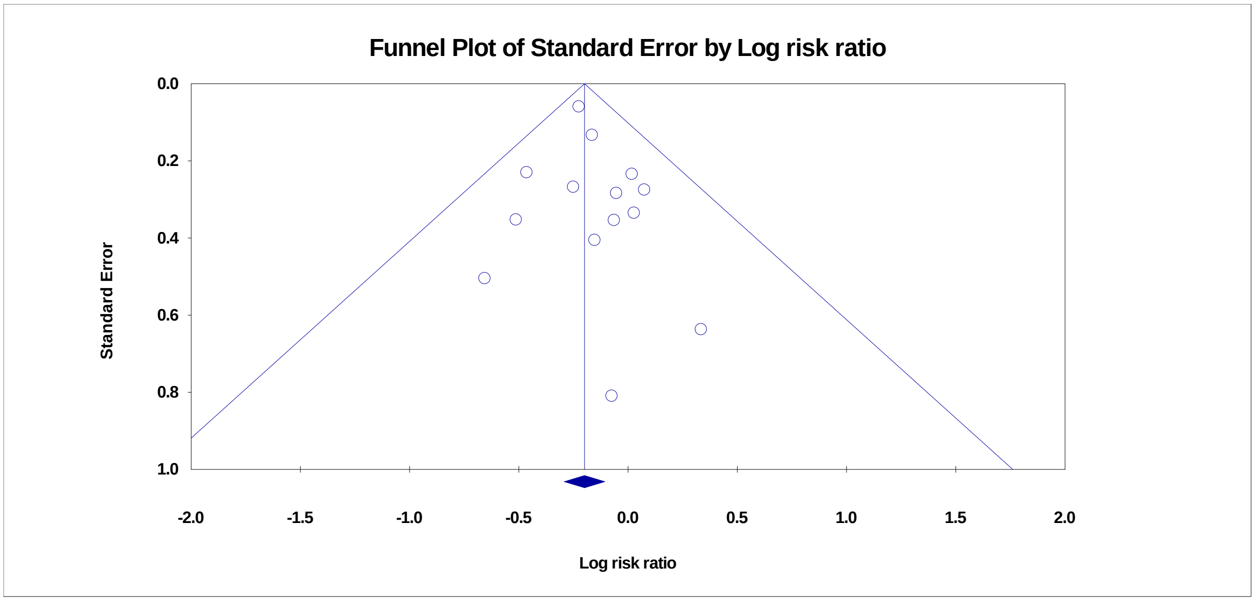

Supplemental Figure 6. Funnel plot for the effect of prenatal multiple micronutrient supplements on the small vulnerable newborn type of preterm-AGA-LBW.

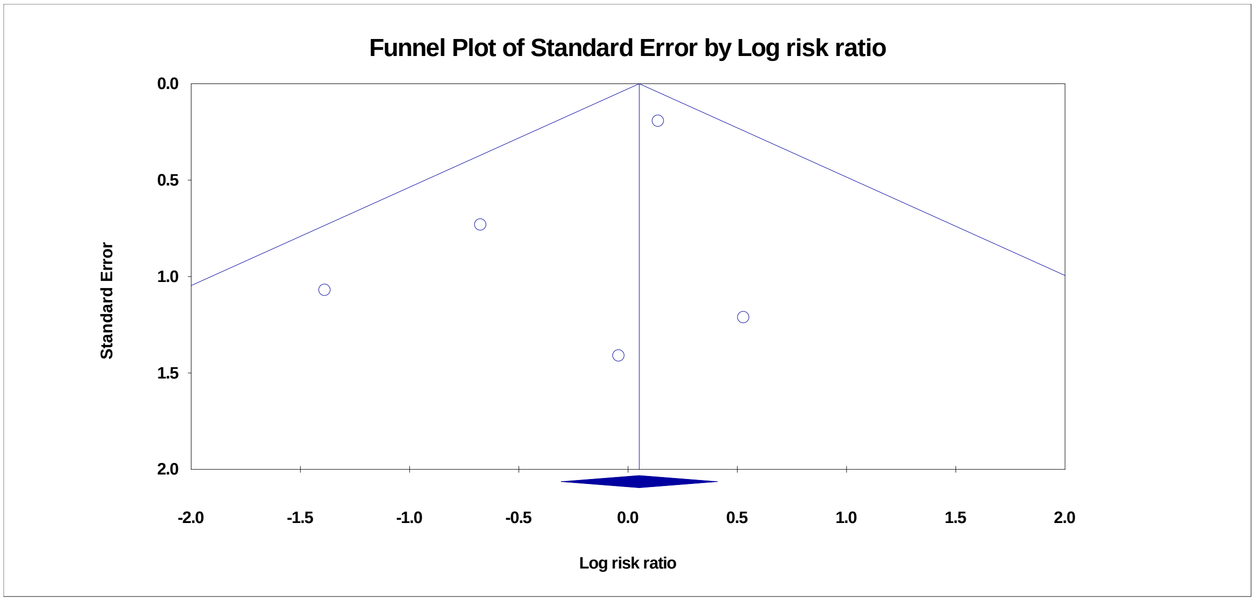

Supplemental Figure 7. Funnel plot for the effect of prenatal multiple micronutrient supplements on the small vulnerable newborn type of term-AGA-LBW.

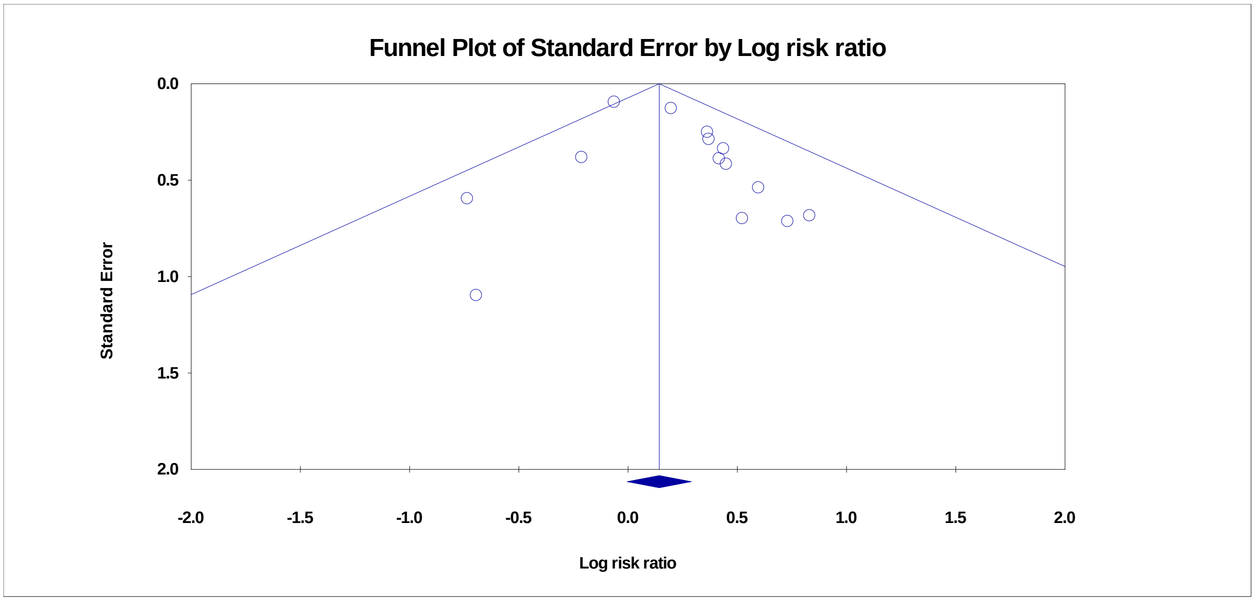

Supplemental Figure 8. Funnel plot for the effect of prenatal multiple micronutrient supplements on the small vulnerable newborn type of term-LGA-nonLBW.

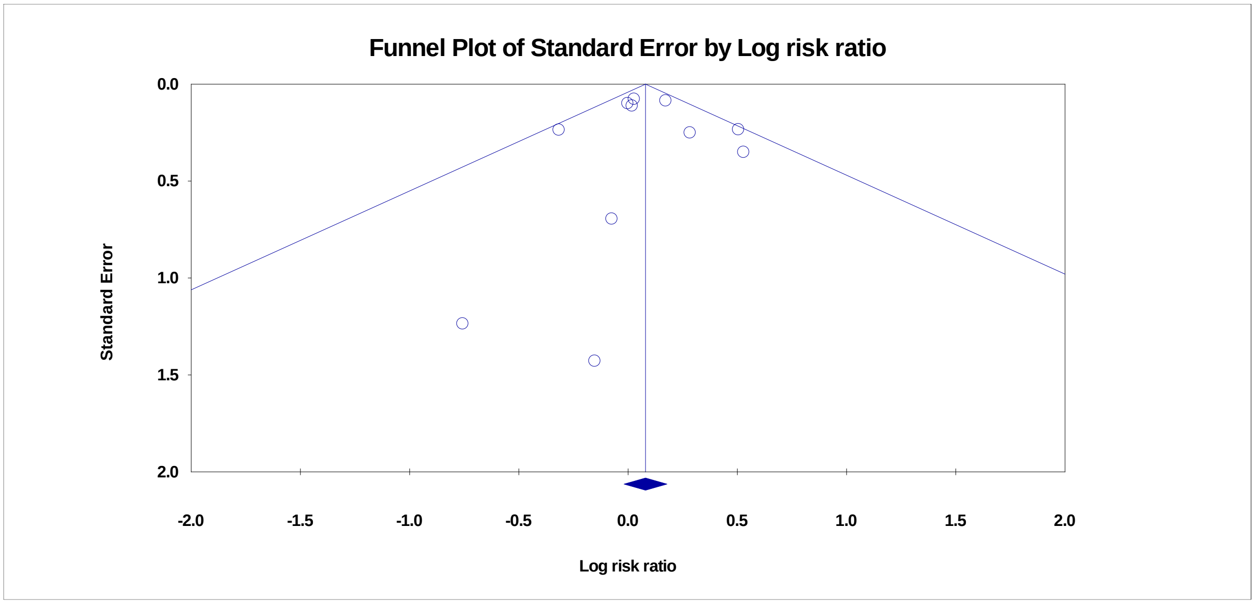

Supplemental Figure 9. Funnel plot for the effect of prenatal multiple micronutrient supplements on the small vulnerable newborn type of preterm-LGA-nonLBW.

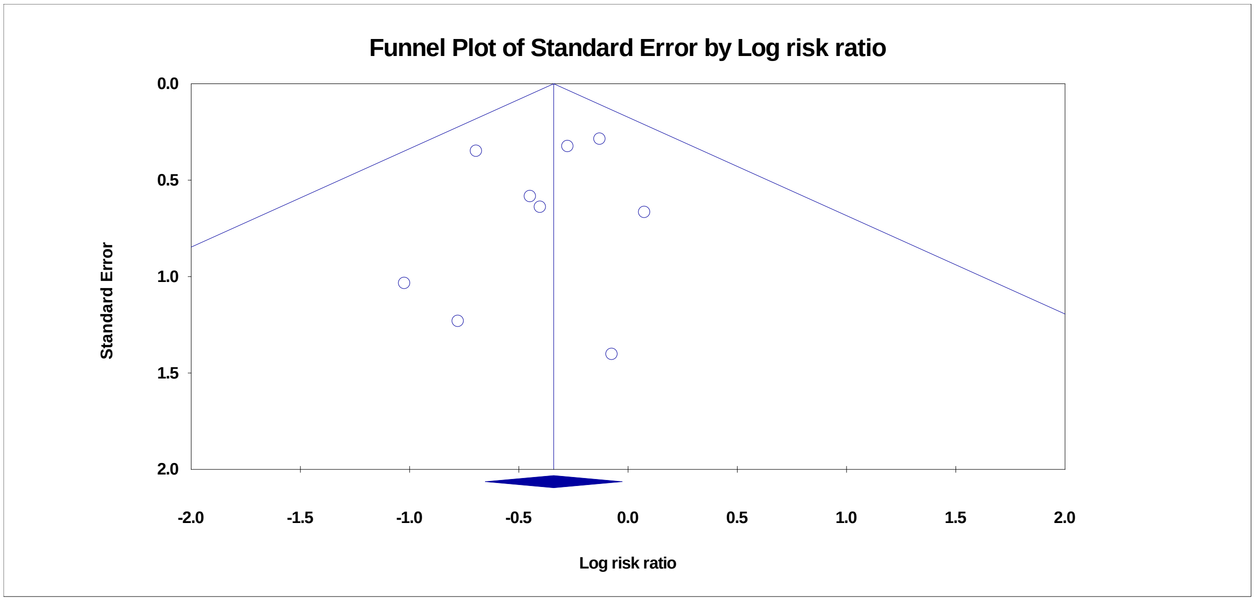

Supplemental Figure 10. Funnel plot for the effect of prenatal multiple micronutrient supplements on the small vulnerable newborn type of preterm-LGA-LBW.

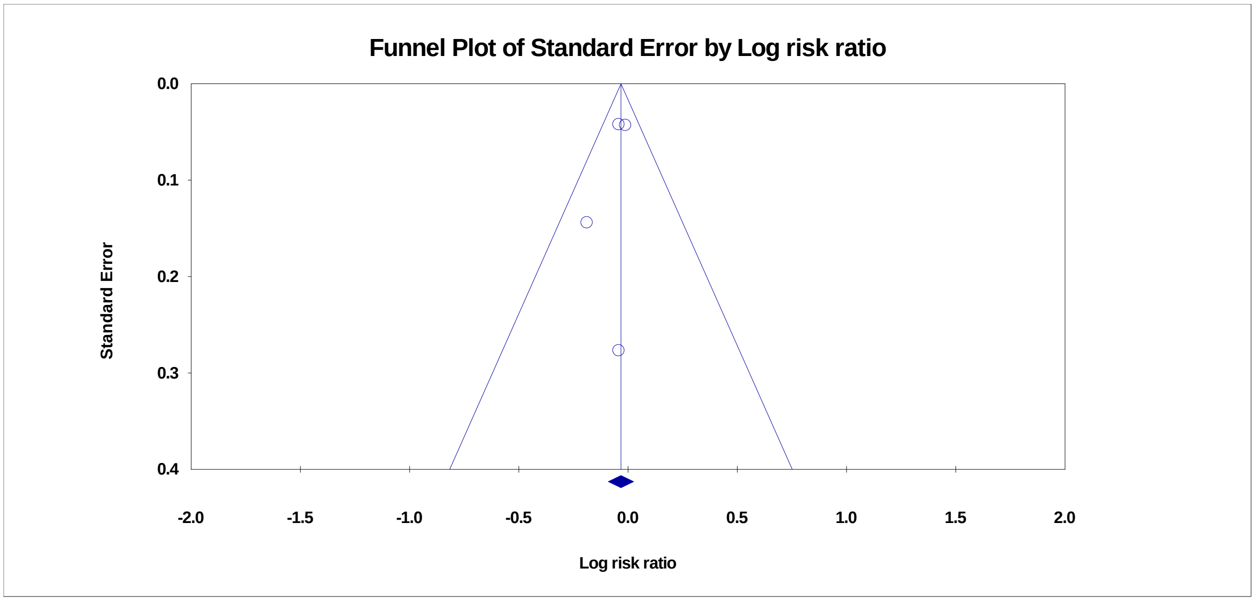

Supplemental Figure 11. Funnel plot for the effect of prenatal small-quantity lipid-based nutrient supplements on the small vulnerable newborn type of term-SGA-nonLBW.

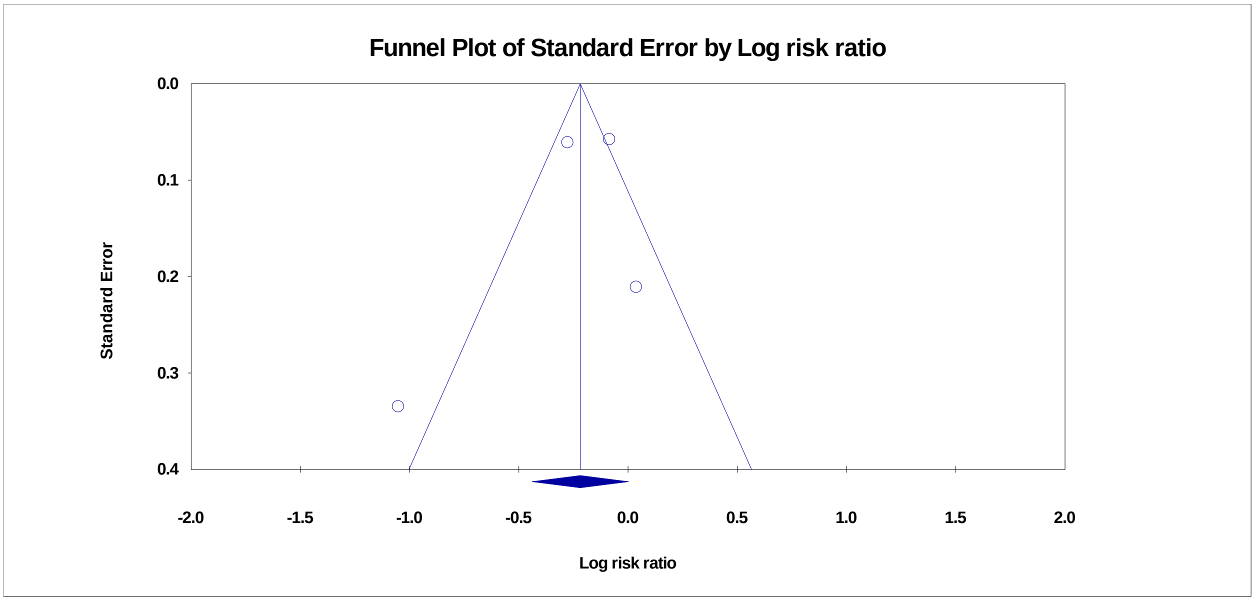

Supplemental Figure 12. Funnel plot for the effect of prenatal small-quantity lipid-based nutrient supplements on the small vulnerable newborn type of term-SGA-LBW.

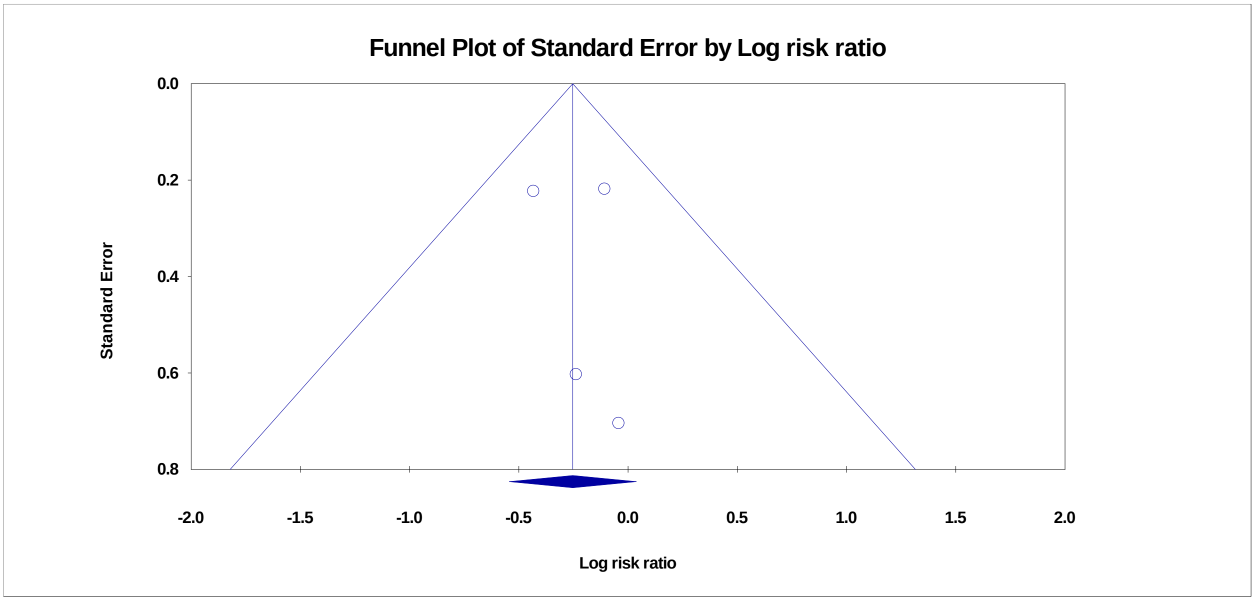

Supplemental Figure 13. Funnel plot for the effect of prenatal small-quantity lipid-based nutrient supplements on the small vulnerable newborn type of preterm-SGA-LBW.

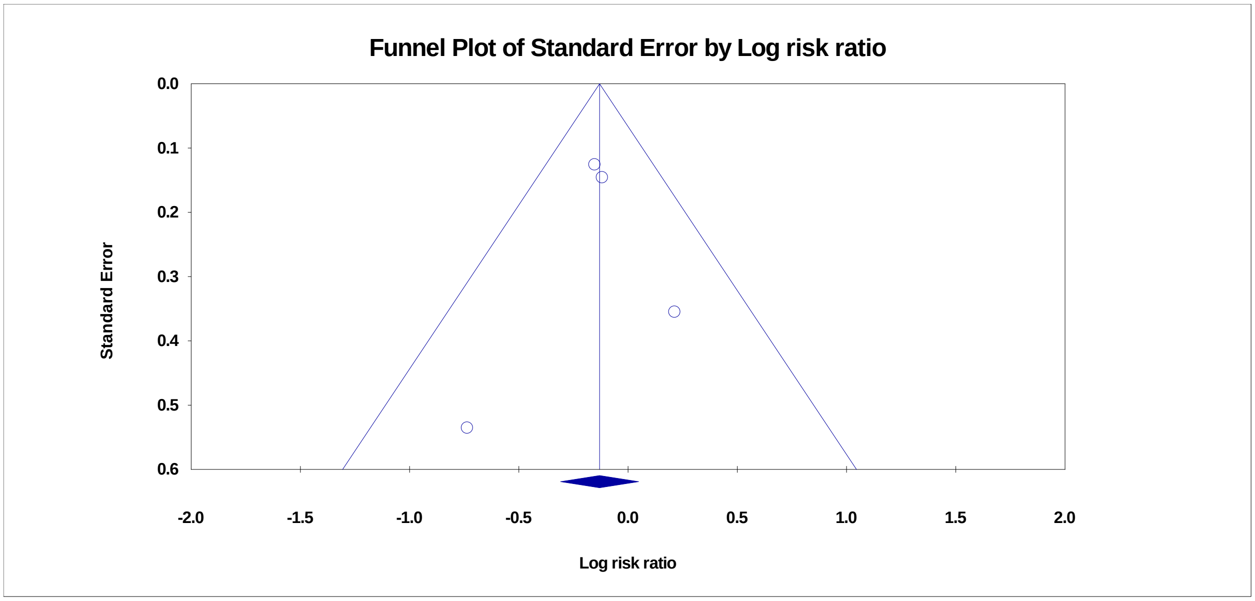

Supplemental Figure 14. Funnel plot for the effect of prenatal small-quantity lipid-based nutrient supplements on the small vulnerable newborn type of preterm-AGA-nonLBW.

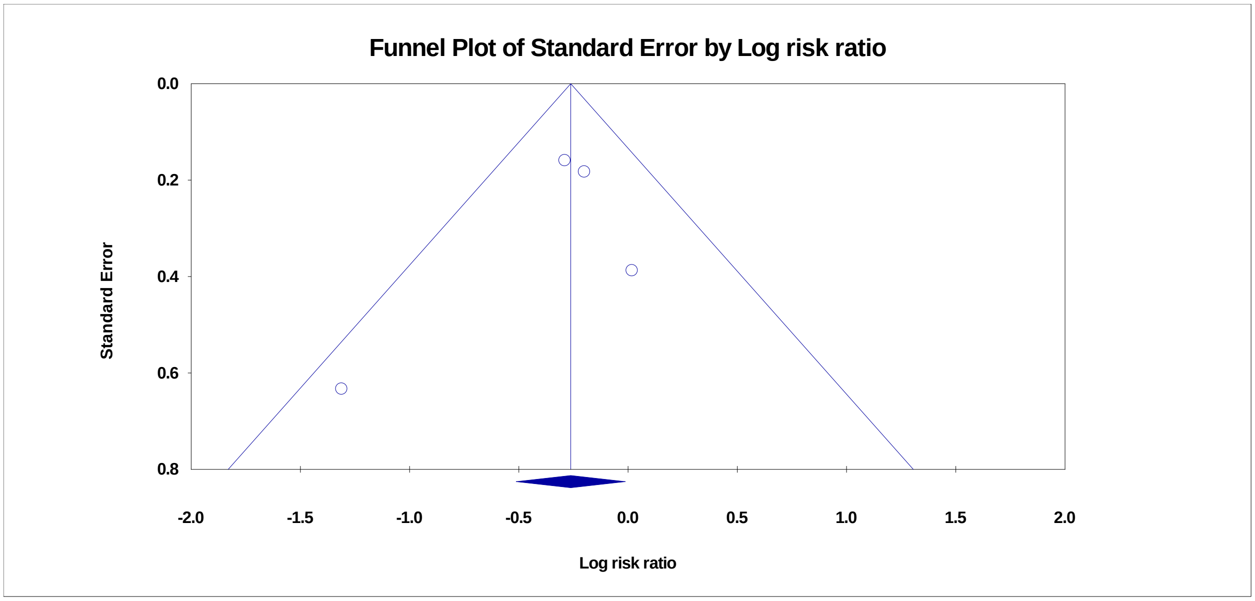

Supplemental Figure 15. Funnel plot for the effect of prenatal small-quantity lipid-based nutrient supplements on the small vulnerable newborn type of preterm-AGA-LBW.

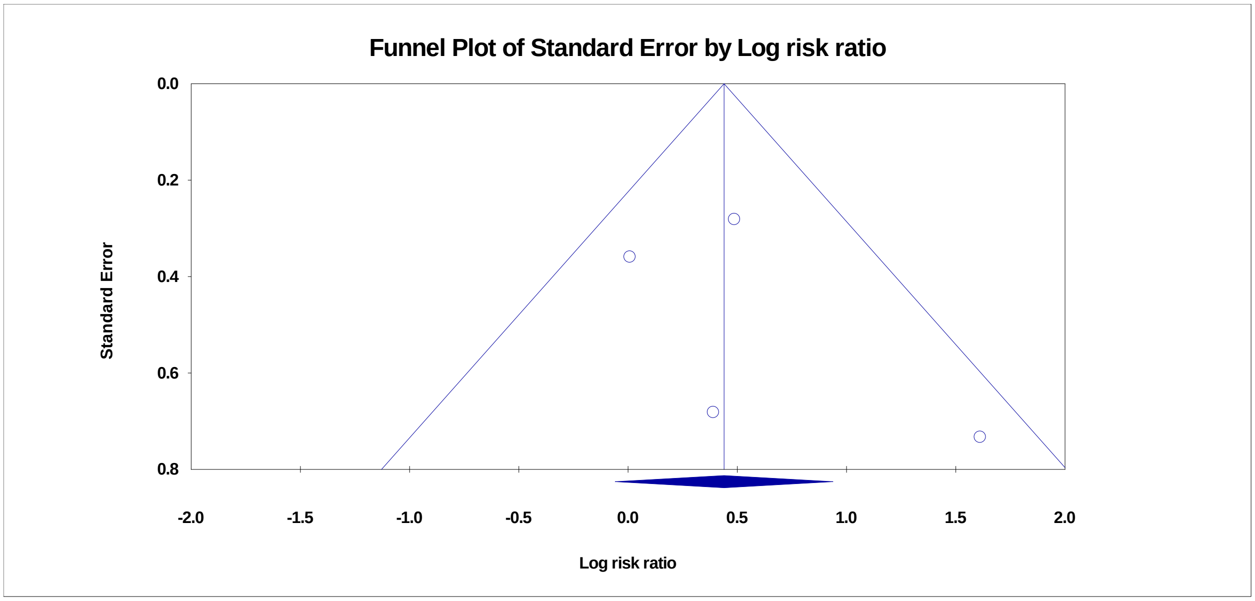

Supplemental Figure 16. Funnel plot for the effect of prenatal small-quantity lipid-based nutrient supplements on the small vulnerable newborn type of term-LGA-nonLBW.

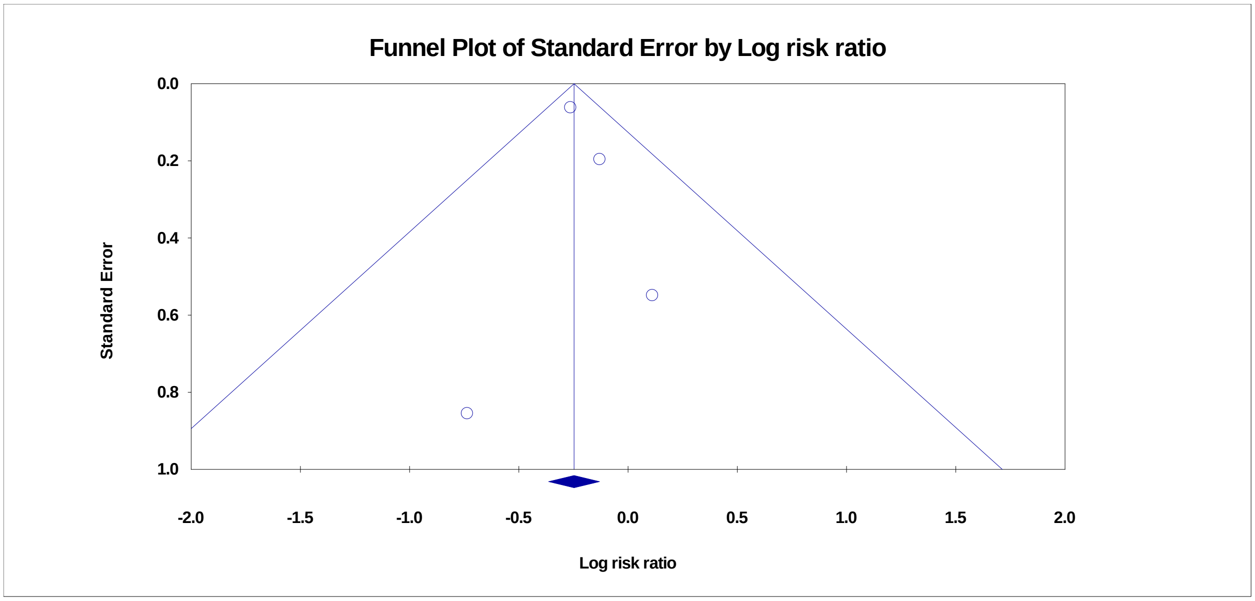

Supplemental Figure 17. Funnel plot for the effect of prenatal small-quantity lipid-based nutrient supplements on the small vulnerable newborn type of preterm-LGA-nonLBW.

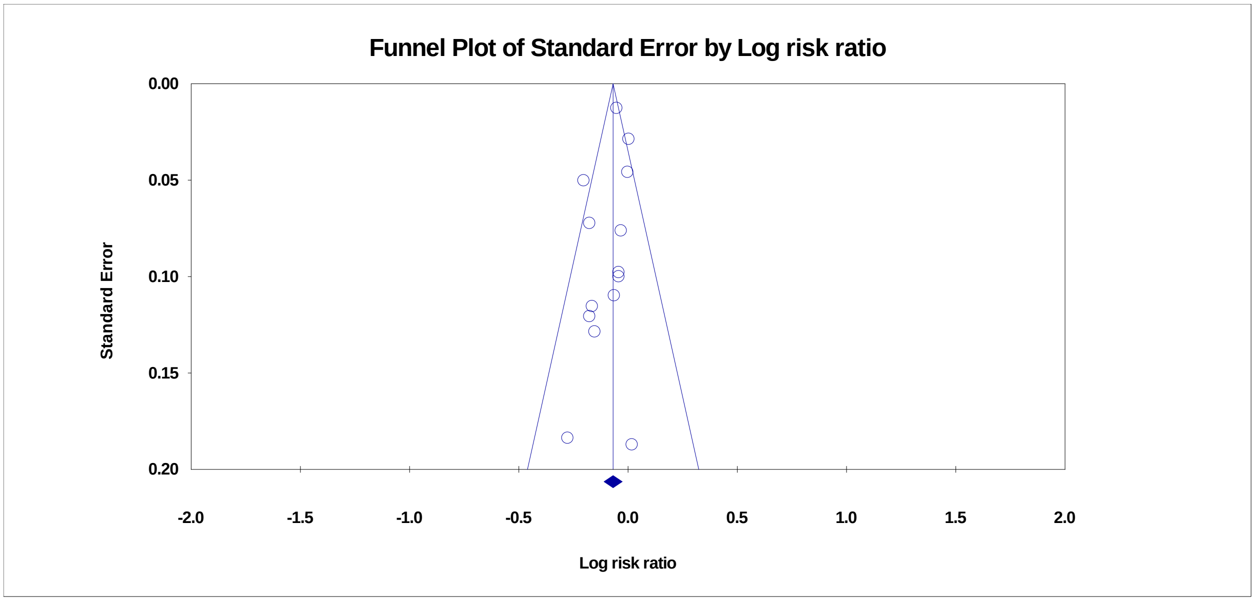

Supplemental Figure 18. Funnel plot for the effect of prenatal multiple micronutrient supplements on the small vulnerable newborn type of term-SGA.

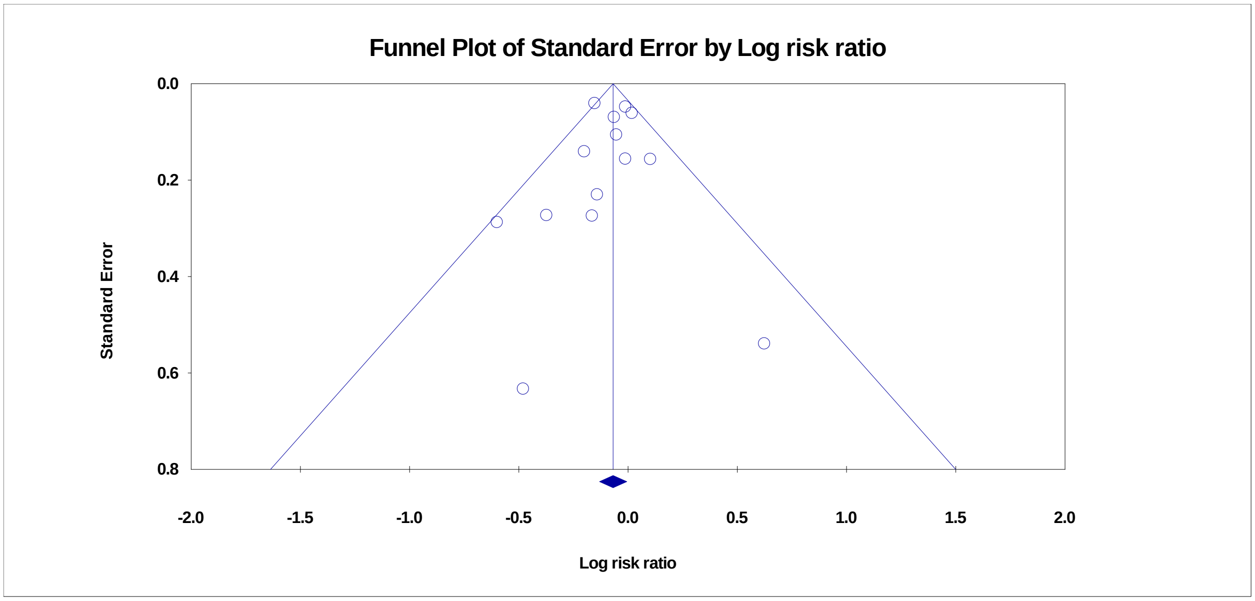

Supplemental Figure 19. Funnel plot for the effect of prenatal multiple micronutrient supplements on the small vulnerable newborn type of preterm-nonSGA.

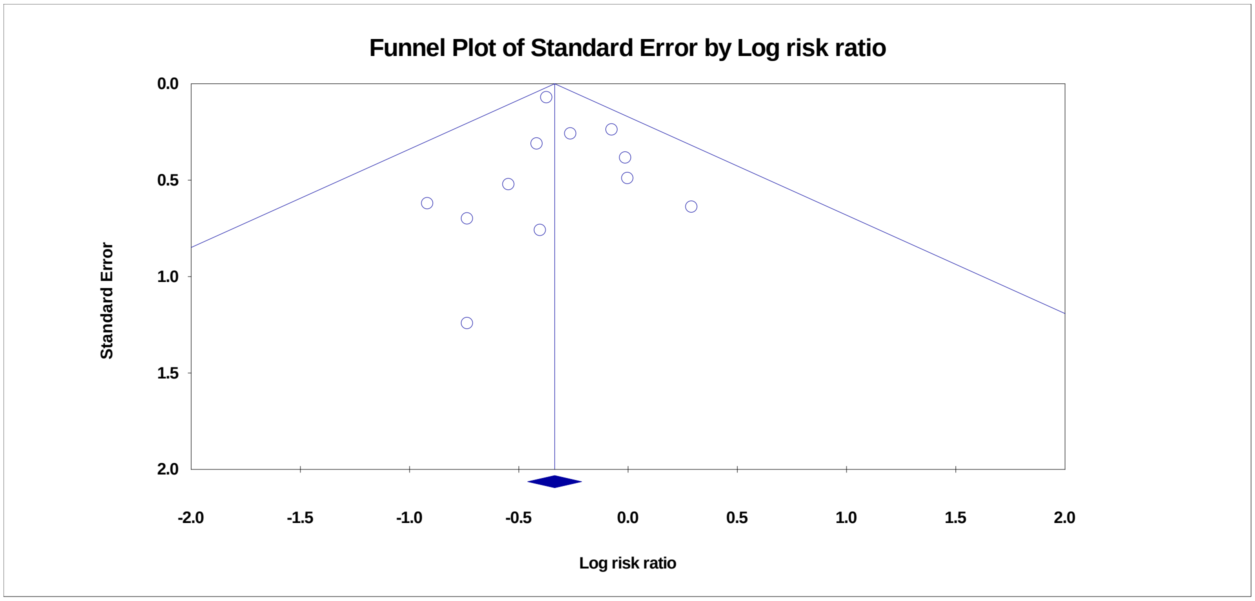

Supplemental Figure 20. Funnel plot for the effect of prenatal multiple micronutrient supplements on the small vulnerable newborn type of preterm-SGA.

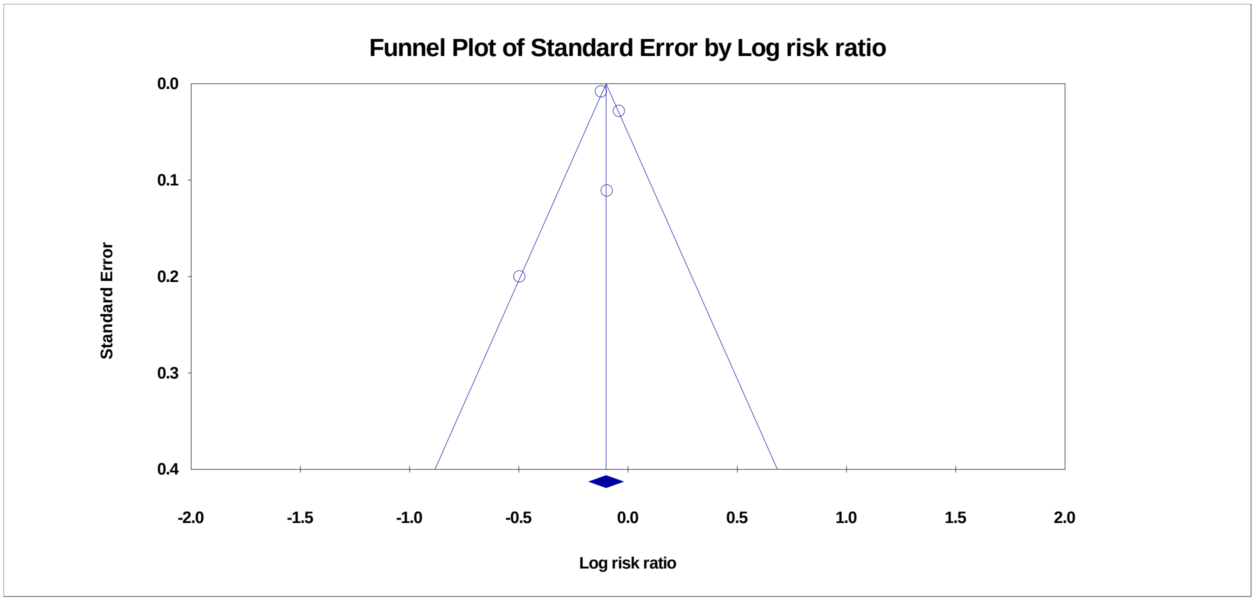

Supplemental Figure 21. Funnel plot for the effect of prenatal small-quantity lipid-based nutrient supplements on the small vulnerable newborn type of term-SGA.

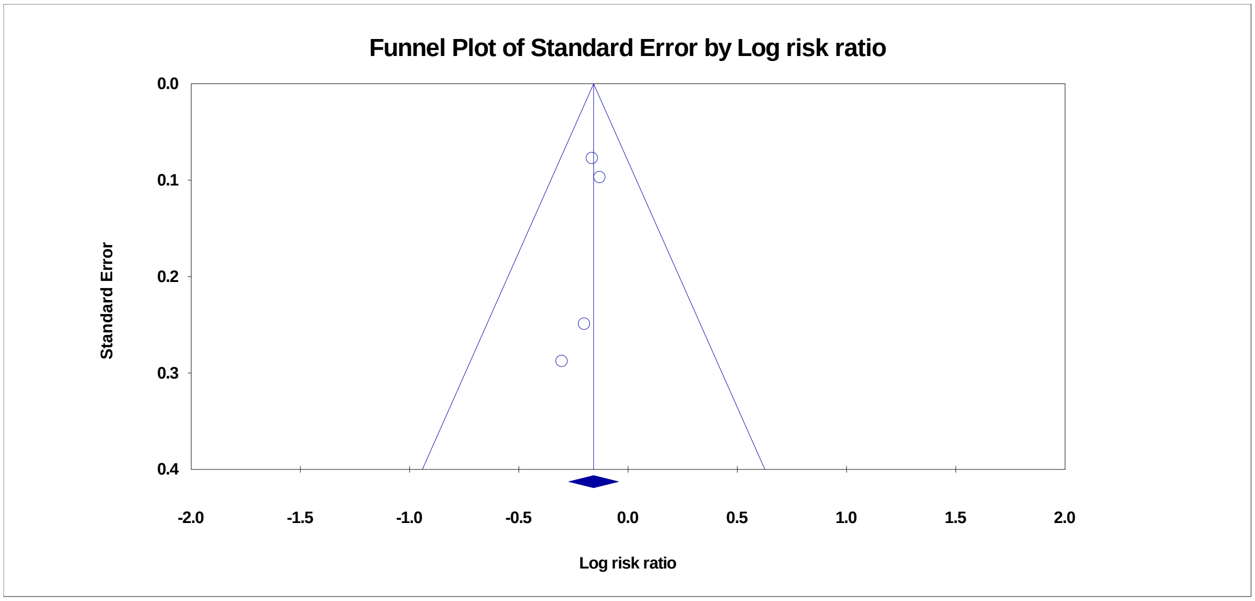

Supplemental Figure 22. Funnel plot for the effect of prenatal small-quantity lipid-based nutrient supplements on the small vulnerable newborn type of preterm-nonSGA.

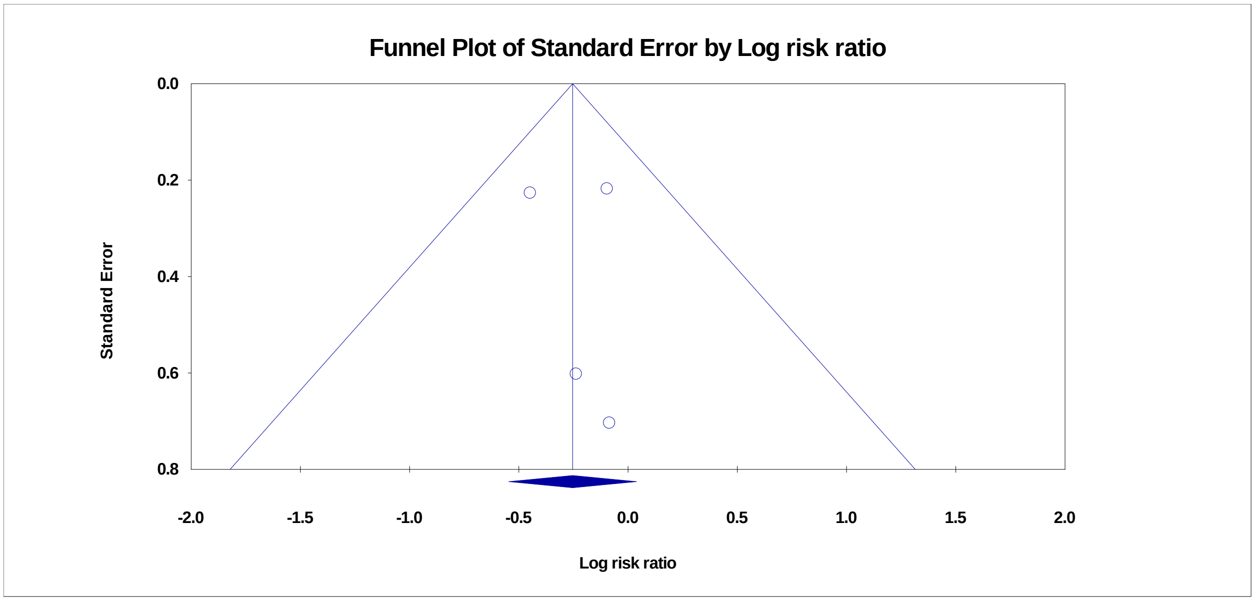

Supplemental Figure 23. Funnel plot for the effect of prenatal small-quantity lipid-based nutrient supplements on the small vulnerable newborn type of preterm-SGA.

References

1. Kaestel P, Michaelsen KF, Aaby P, Friis H. Effects of prenatal multimicronutrient supplements on birth weight and perinatal mortality: a randomised, controlled trial in Guinea-Bissau. *Eur J Clin Nutr* 2005; **59**(9): 1081-9.
2. Zagré NM, Desplats G, Adou P, Mamadoultaibou A, Aguayo VM. Prenatal multiple micronutrient supplementation has greater impact on birthweight than supplementation with iron and folic acid: a cluster-randomized, double-blind, controlled programmatic study in rural Niger. *Food Nutr Bull* 2007; **28**(3): 317-27.
3. Shankar AH, Jahari AB, Sebayang SK, et al. Effect of maternal multiple micronutrient supplementation on fetal loss and infant death in Indonesia: a double-blind cluster-randomised trial. *Lancet* 2008; **371**(9608): 215-27.
4. Sunawang, Utomo B, Hidayat A, Kusharisupeni, Subarkah. Preventing low birthweight through maternal multiple micronutrient supplementation: a cluster-randomized, controlled trial in Indramayu, West Java. *Food Nutr Bull* 2009; **30**(4 Suppl): S488-95.
5. Hanieh S, Ha TT, Simpson JA, et al. The effect of intermittent antenatal iron supplementation on maternal and infant outcomes in rural Viet Nam: a cluster randomised trial. *PLoS Med* 2013; **10**(6): e1001470.
